# Modification and validation of the InVEST erosion model for application at a national scale

**DOI:** 10.1371/journal.pone.0353736

**Published:** 2026-07-17

**Authors:** Danny A.P. Hooftman, Richard F. Pywell, Paul M. Evans, Cecily E.D. Goodwin, John W. Redhead, Lucy E. Ridding, Varun Varma, James M. Bullock

**Affiliations:** 1 Lactuca, Environmental Data Analyses and Modelling, Diemen, The Netherlands; 2 UK Centre for Ecology & Hydrology, Wallingford, United Kingdom; 3 Rothamsted Research, Harpenden, United Kingdom; Acharya Brojendra Nath Seal College, INDIA

## Abstract

Soil erosion is an important ecological impact of human activities such as agriculture, and models can aid in identifying areas most at risk. However, to aid targeting of actions, predictions must simultaneously cover large spatial extents and account for site-specific variation in retention, while achieving adequate parameterisation and evaluation of models remains demanding. To address these issues, we adapted and assessed an erosion model using the InVEST platform, which applies the Revised Universal Soil Loss Equation (RUSLE), for Great Britain (GB). We parametrised the model using GB-specific input data, for multiple crop types, and integrating new factors including sub-annual periodicity, a GB-specific erosivity layer, and Normalized Difference Vegetation Index (NDVI) derived cover management metrics. Our modelled predictions validated well against sediment concentration measurements in rivers, with a predictive accuracy of 78% on normalised data and rank correlation of 0.46 between predictions and observations. However, absolute model values were overestimated by 28-times, especially at higher levels of sediment erosion. This seems related to a lack of measurements of sediment concentrations at peak flows in the validation data, combined with known RUSLE methodological issues. To allow for future model training, we call for improvements to national sediment load monitoring in rivers, by including measurements during peak flows especially, and inclusion of a within stream sedimentation function in the InVEST Sediment Delivery Ratio model. Our InVEST model parametrisation example provides a valuable tool for relative risk mapping, comparing regions and targeting where ecosystems are more prone to erosion. Cover management factors based on NDVI observations are a substantial methodological improvement for RUSLE modelling that can be readily reproduced in other locations. Whereas a sub-annual version did not lead to differences from an annual model in this all-year around rain area it could enhance modelling for more seasonal areas.

## 1. Introduction

Healthy soils are the foundation of agriculture and have been an essential resource in supporting human needs through history [1,2]. Soil erosion is the process of topsoil loss, and although a natural process, is exacerbated by many agricultural activities [3], which have become more intensive over the 21^st^ century [4–6]. Increased soil erosion negatively affects ecosystem functioning and disrupts natural processes such as vegetation production, water storage and filtration, nutrient cycling, biodiversity, and carbon storage [7,8]. In agricultural areas, topsoil erosion reduces potential crop or grazing areas and depletes nutrients for future production [9]. Consequently, erosion has become one of the major threats to soils, and therefore agriculture, worldwide [6,7,10,11].

The costs of erosion can be substantial: for instance, €2.3 Billion annually across the European Union (EU) because of water erosion [12]. With soil loss by water erosion projected to increase by 20% in 2050 [11], the EU has listed soil erosion among the key threats within the Soil Thematic Strategy of the European Commission [7,13,14]. It is estimated that in the United Kingdom (UK) alone, over 2-million hectares of soil are at risk of erosion, with the loss of 40% to 60% of the organic carbon of arable soils being attributed to intensive agriculture [15]. Graves and co-workers [16] estimated soil degradation costs for England and Wales from £0.9 to £1.4 Billion per year.

Predictive soil erosion models can aid in identifying areas that are most vulnerable to soil erosion, estimating potential erosion rates, and highlighting causes of this erosion. Existing models range from simple empirical and conceptual models to complex physics-based mathematical models [3,17]. Karydas and co-workers [18] identified 82 distinct types of soil-erosion models, a number which continues to grow [5,19]. These contain different combinations of equations, running over different spatial & temporal scales, and with various levels of complexity. Within these, the dominant equational framework to predict erosion is the Universal Soil Loss Equation (USLE) [3,20] and its Revised form (RUSLE) [21]. (R)USLE estimates a long-term average for annual soil loss by rainfall and overland erosion [7].

One widely-used modelling platform that provides a RUSLE-based approach is InVEST [22] (“INtegrated Valuation of Ecosystem Services and Trade-offs”), one of the world’s leading platforms for Ecosystem Service (ES) estimations [23,24]. The RUSLE-based InVEST Sediment Delivery Ratio (SDR) model is a spatially explicit erosion model focussing on overland erosion and soil retention by vegetation, i.e., avoided erosion. Outputs from the model include the realised sediment export delivered to streams, as well as the amount of sediment retained by vegetation and tillage management. InVEST is an open-source model framework, integrating a wide range of ES models, including for sediment and nutrient retention and loss [25]. The models are especially designed for ease of use in data-poor regions, serving a large global community. InVEST is unique in that it allows user*-*defined input data for all model parameters, in contrast to other ES model frameworks such as ARIES or Co$ting Nature, which draw in pre-existing datasets [24,26]. In InVEST, therefore, the extent and quality of inputs is up to the user, making the models extremely flexible, but also making an adequate parameterisation strategy paramount. Hence, to ease use in data-poor areas, parametrisation strategies need to be developed in data-rich areas which facilitate validation of results [24,27,28].

Our test region, Great Britain (GB), is a useful example of a data-rich region for model parameterising and validation. In the GB context, other InVEST models have been well established, parameterised, and evaluated, e.g., in [29–31]. To our knowledge, no sediment retention or erosion predictions have been developed for any substantial part of GB while accounting for periodic variation in retention by vegetation and incorporating the detailed GB land cover maps available [32,33]. Though, Europe-wide annual predictions, including the UK, have been published using broader-scale datasets as inputs, such as Corine land cover [7,11]. The absence of such assessments is surprising for such an important environmental concern for which model outputs are key in policy relative priority analyses [11,34,35]. For example, models can highlight those agricultural areas where retention capacity needs to be improved, especially when parameterised with local data, which reduces the inherent inaccuracies of global and other large-scale data [36]. Therefore, GB serves as useful focus for developing an exemplar parameterising strategy for a data-rich area.

Furthermore, there is a need to include within-year variation in erosion estimates [3,30,37]. Sediment run-off is not continuous but occurs during erosive heavy rainfall events. Agricultural crops also vary in cover throughout the year, which impacts erosivity. Periods of high rainfall may coincide with times of low vegetation cover (e.g., winter in GB’s temperate climate) and high vegetation cover could coincide with drier periods (e.g., GB’s summer). Therefore, as these fluctuations in erosive pressure through rainfall and soil cover vary throughout the year, the estimated cumulative annual sum of sediment lost could be different compared to one modelled using averaged per year parameter values.

In this work, we present a GB assessment of the amount of estimated erosion and retention. We parametrise the SDR InVEST model, driven by local data, and incorporate a new seasonally periodic approach to the model. Periodic GB-specific cover management parameters (*C-*factors) for eleven crop types are estimated based on observed satellite Normalized Difference Vegetation Index (NDVI) values following [25]: a new approach in sediment flux modelling. The developed model estimates are validated against suspended solid concentrations (sediment) in rivers throughout England and Wales.

## 2. Methods

Calculations were performed using InVEST v3.13.0, whilst all pre- and post data-processing was completed using ArcGIS Pro v3.6.1 and Matlab v9.14.0. Procedures are in *italics*, and they refer to ArcGIS unless noted as Matlab. All computer codes used in processing can be found on GitHub.

**Fig 1:**
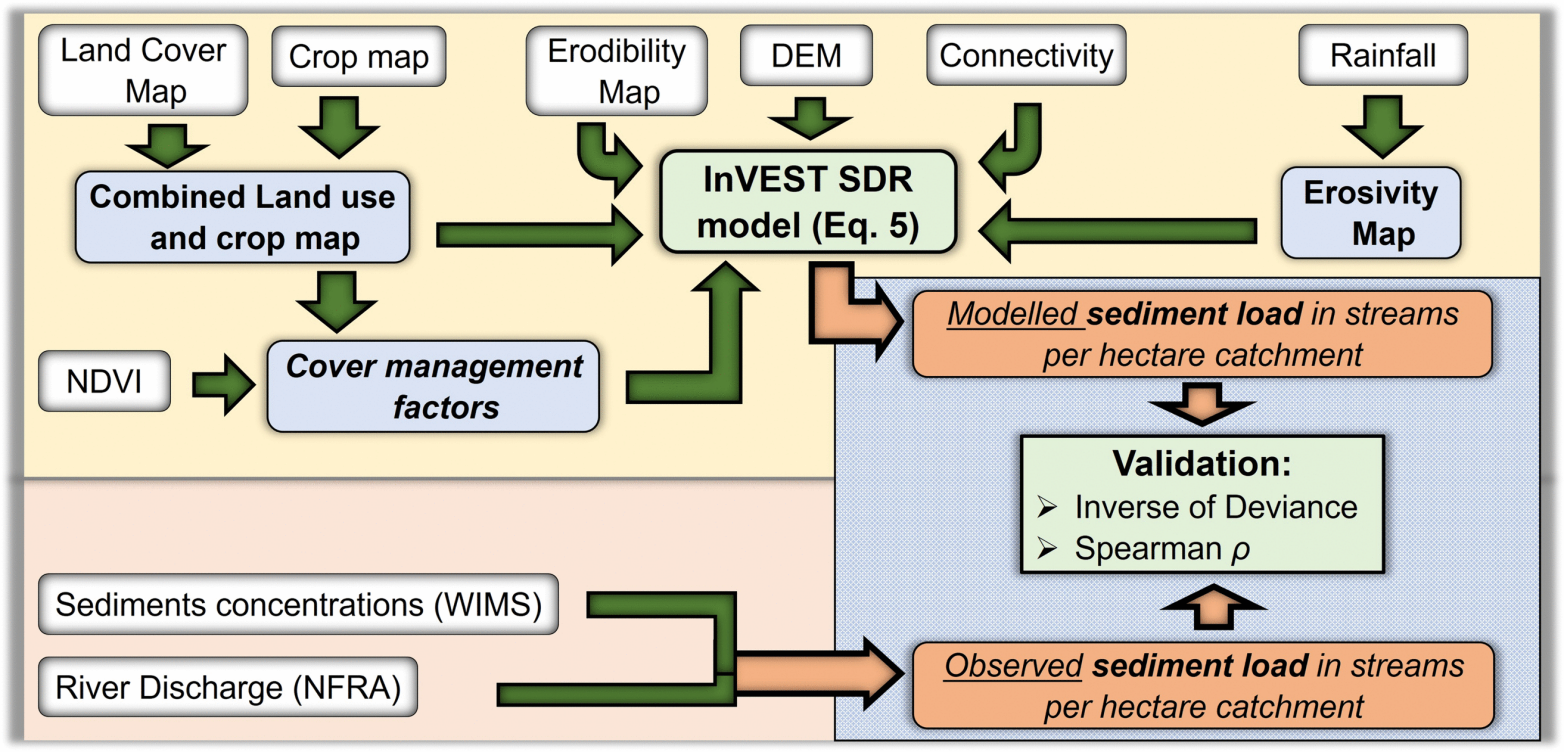
Analyses flow chart, including the inputs into the here generated new approach inputs in blue: the combined land use map, the erosivity layer and the observation-based cover management factors (2.3.1).

**Fig 2:**
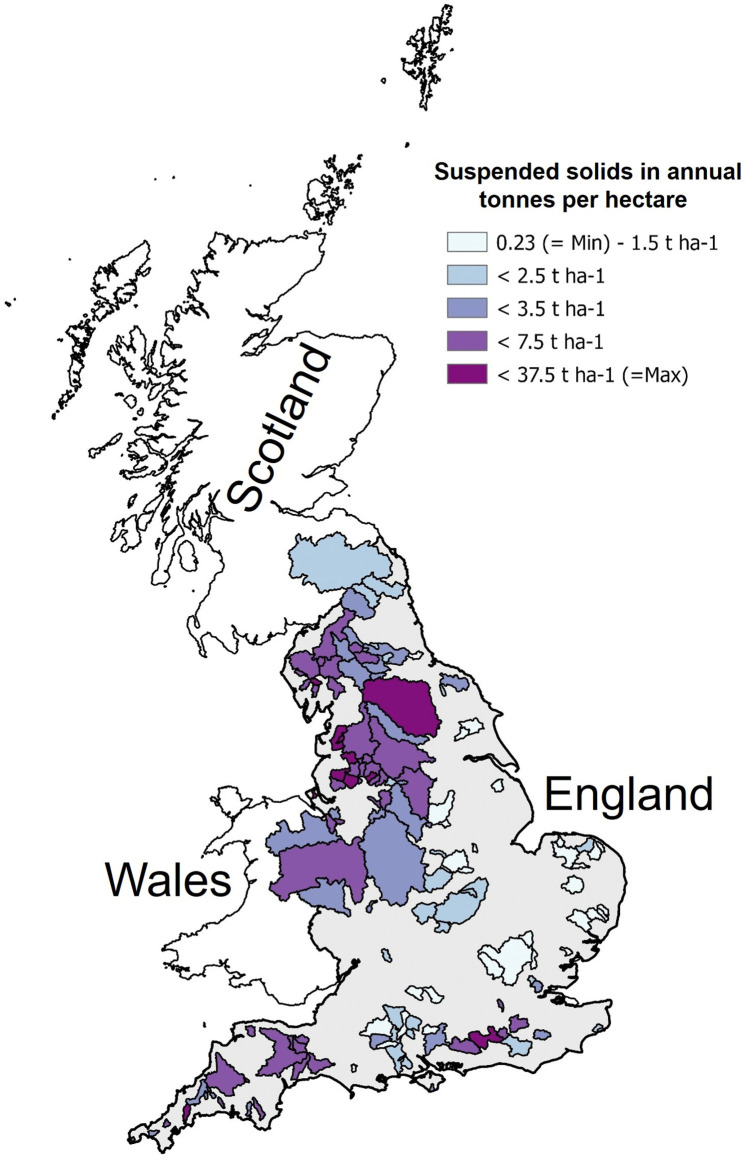
The 178 catchments used for validation. Provided is the mean annual amount of concentrations of suspended solids (‘sediment’) from WIMS [40] multiplied with river discharge from NFRA [41]. Indicated are the GB nations in this study: England (in grey), Scotland & Wales. UK shape licensed under CC BY 4.0 [42].

In Fig 1, we depict the analysis as flow chart. The area of study is Great Britain (GB), encompassing the nations of England, Scotland & Wales in the United Kingdom (Fig 2). GB is 209,331 km² in size and encompasses 92% of the UK by area. All geographic datasets were reprojected to the British National Grid (EPSG 27700; OSGB36), where applicable using the ArcGIS *cubic-resampling* procedure. The InVEST SDR model runs, by default, at the spatial resolution of the input Digital Elevation Model raster (DEM; here 50 × 50 meters), with all other data resampled and aligned to that DEM within the InVEST module. As InVEST transfers values and does not recalculate in resampling, input data that are in tonnage per hectare need to be recalculated to the DEM resolution when summed (such as the K-raster, a division by four). Data for 2015, or as close to as possible, were used as far as feasible to align with other mapping activities under the overarching project, including [38,39].

### 2.1. The InVEST SDR model

The InVEST SDR model predicts the cumulative amount of erosion from fields that reaches the streams. It is a spatially explicit model focussing on overland erosion, and does not model gully, bank, or mass erosion [22]. The amount of annual sediment loss in metric tonnes (t) ha^-1^ yr^*-*1^ is calculated by the Revised Universal Soil Loss Equation (RUSLE) [3,7,21].

The general RUSLE equation for a gridcell *i* follows:


$$\begin{array}{c} A_{i}=R_{i}\times K_{i}\times L_{i}\times S_{i}\times C_{i}\times P_{i} \end{array}$$ (1)

where:

*A* is the mean annual soil loss (t sediment ha^-1^ yr^*-*1^);

*R* is rainfall erosivity ([MJ mm^-1^] ha^-1^ h^*-*1^ yr^*-*1^; see 2.3.1);

*K* is soil erodibility (t h^*-*1^ ha^-1^ per [MJ mm^-1^ ha^-1^] = t h^*-*1^ per [MJ mm^-1^]; see 2.3.2);

*L* is the slope length factor (unitless; see [3, 22]);

*S* is the slope gradient factor (unitless; see [3, 22]);

*C* is the cover management factor (unitless; see 2.3.1);

*P* is the support practice factor (unitless; see 2.3.1).

A description and the full mathematical framework of the RUSLE approach in InVEST is provided in the InVEST user manual [22]. The model estimates the proportion of sediment eroded per gridcell *i* that reaches the streams: the Sediment Delivery Ratio (SDR), ranging from 0–1. This SDR is derived from slope characteristics obtained from a DEM, incorporating direction and aspect, and combined with connectivity parameters (2.3.2). Slopes are characterised by *L* and *S* as inputs in equation (1); a combination referred to as the slope length-gradient (*LS*) factor. See [22,43] for further detail on the *LS*-factor, with [43] providing a step-wise calculation for an EU-wide estimate of erosion in Europe [7]. Higher SDR values indicate that a greater fraction of sediment eroded from an uphill gridcell is delivered to a downslope stream. Subsequently, realised sediment export (*E*) from a gridcell *i* that reaches the streams is calculated as:


$$\begin{array}{c} E_{i}=A_{i}\times {SDR}_{i} \end{array}$$ (2)

With SDR ratio for a pixel derived from the conductivity index *IC*_*i*_ following [22]:


$$\begin{array}{c} {SDR}_{i}=\frac{{SDR}_{max}}{\text{1}+e^{\left(\frac{\left(IC\text{0}-IC\text{1}\right)}{k}\right)}} \end{array}$$ (3)

with *SDR*_*max*_ the maximum theoretical *SDR; IC*_*0*_ and *k* connectivity parameters (2.3.2).

Model outputs used in this study included (a) the realised sediment load that reaches the streams in metric tonnes sediment hectare^-1^ yr^*-*1^ for each pixel (*E*_*i*_, equation 2) (b) the potential sediment loss that has been captured by vegetation and management features per pixel (*V*_*i*_). *V* is the absolute difference between *A* calculated with- and without cover management factors *C* and *P* included (see equation 1).

Avoided erosion (*V*_*i*_) is dependent on the absolute amount of erosion. If there is low potential erosion the absolute avoided value is necessarily low, whereas when potential erosion is high the absolute avoided erosion could be high. Vegetation type is known to influence the potential for erosion, and this is represented using the *C-*factor (equation 1). Therefore, as additional output metric, we assessed the relative role of vegetation in erosion. We calculated the fraction of erosion avoided for gridcell *i* capturing the proportional effect of vegetation and cover management ($\hat{V_{i}}$) using:


$$\begin{array}{c} \hat{V_{i}}=\;\left(\text{1}-\left[\frac{V_{i}}{A_{i}}\right]\right) \end{array}$$ (4)

### 2.2. Periodicity

Following equation (1), the mean soil loss (*A*_*i*_) can be calculated as an annual value, and this is how InVEST is typically run. To generate periodicity, we suggest that equation (1) could be split into shorter periods, allowing the incorporation of periodic *C-* and *R-*factors (2.3.1). We reason that the *R-*factor is defined as the sum of all erosive events (*f*) throughout the year that exceed a threshold of rainfall intensity and length [3,21]. Therefore, the RUSLE equation for InVEST (equation 1) could be rewritten as the sum of all erosive rainfall events over a specified period for gridcell *i* with a corresponding period-specified cover management factor (*C*_*ij*_) from the prevailing vegetation type during these rainfall events as:


$$\begin{array}{c} A_{i}=\left(\sum_{f=\text{1}}^{n}R_{if}\right)\times K_{i}\times L_{i}\times S_{i}\times \left(\frac{\sum_{f=\text{1}}^{n}C_{if}}{n}\right)\times P_{i} \end{array}$$ (5)

Where parameters are as in equation (1), *f* is an erosive event, and *n* is the total number of erosive events.

Extending this reasoning, $\sum_{f=\text{1}}^{n}R_{if}$ could denote the annual sum of individual erosive events but is actually a sum of events within any period, so that in each period *j* it is the sum of events *f* within it. Letting *j* be a period of events *f*, Equation (5) can be rewritten as:


$$\begin{array}{c} A_{i}=\sum_{\hat{j}=\text{1}}^{n}\left(R_{ij}\times K_{i}\times L_{i}\times S_{i}\times C_{ij}\times P_{i}\right) \end{array}$$ (6)

Equation (6) forms the basis for splitting the calculation into 23 intervals of 16 days, each with period-specific *R-* and *C-*values. These intervals correspond to these approximate half-month observations of NDVI below (2.3.1). To do so, InVEST was run 23-times, each with the set of *C-* and *R-*values for that interval, with all other input parameters kept constant. As in each run, the *R*-layer provides the respective fraction of total *R* based on rainfall (see 2.3.1), a periodic run represents the occurring erosion within that period. InVEST does not contain a time axis but is a single linear calculation of RUSLE with *R* being the only temporal component through the length of the period used to sum erosive events. Therefore, InVEST is not necessarily an annual model but is applicable to any length of time specified by the period covered by *R*_ij_.

Subsequently these 23 runs were summed into one annual figure as $E_{i}=\sum_{j=\text{1}}^{\text{2}\text{3}}E_{ij}$ and $V_{i}=\sum_{j=\text{1}}^{\text{2}\text{3}}V_{ij}$with *j* being the 16-day intervals. As the connectivity, *LS*-factor, and following SDR calculation (equation 3; [22]) do not include *R*_*ij*_ or *C*_*ij*_ factors, but are DEM derived, these factors are constant among all runs.

### 2.3. Parameterisation

InVEST requires a set of user*-*defined inputs to populate equation (6). In Fig 1 we depict the different input parameters. We distinguish between: inputs which we generated for this study using new parameterisation approaches (2.3.1); input datasets and parameters from existing data sources (2.3.2); and coefficient values following guidance in [22] (2.3.3). An overview of data-sets used in this study is found in Table 1.

**Table 1:** Datasets used in this study, sources, and licence information.

Dataset	Source	Licence
UK countries shapes extracted from Global Administrative Unit Layers [42]	Food and Agriculture Organization of the United Nations (FAO) Link	Free usage under Creative Commons Attribution 4.0 International
Land Cover Map 2015 (25m raster, GB) [32]	UK-Centre for Ecology and Hydrology Link	Free usage under UKCEH data licence
Land Cover *Plus* Crops 2016, 2017, 2018 & 2019 [33]	UK-Centre for Ecology and Hydrology Link	UKCEH data Licence No. 1668 to Lactuca (DH)
Terra Vegetation Indices 16-Day L3 Global 250m SIN Grid V061 [44]	NASA EOSDIS Land Processes DAAC Link	Free usage under Creative Commons Zero
Copernicus Sentinel-2 NDVI	Google Earth Engine	Free usage under Copernicus Sentinel data licence
Soil erosion by water (RUSLE2015) [7]	European Commission’s Joint Research Centre (JRC) Link	Free usage after data request, required referencing
Soil Erodibility (K- Factor) High Resolution dataset for Europe [35]	European Commission’s Joint Research Centre (JRC) Link	Free usage after data request, required referencing
Rainfall Erosivity in the EU and Switzerland (R-factor) [45]	European Commission’s Joint Research Centre (JRC) Link	Free usage after data request, required referencing
Gridded estimates of daily and monthly areal rainfall for the United Kingdom (1890–2019) [46]	UK-Centre for Ecology and Hydrology Link	Free usage under UKCEH data licence
Digital river network of Great Britain [47]	UK-Centre for Ecology and Hydrology Link	UKCEH data Licence No. 1668 to Lactuca (DH)
Integrated Hydrological Digital Terrain Model [48]	UK-Centre for Ecology and Hydrology Link	UKCEH data Licence No. 1668 to Lactuca (DH)
National River Flow Archive [41]	UK-Centre for Ecology and Hydrology Link	Free usage under NRFA data licence.
UK Water Information Management System [40]	UK Department for Environment, Food & Rural Affairs; Data Services platform Link	Free usage under Open Government Licence v3.0

#### 2.3.1. Datasets using new parameterisation approaches.

*Land Cover Map Plus Crops* (LCM+)*:* The Land Cover Map (LCM), determines the unique land use units for which cover management factors will be assigned. We generated a comprehensive LCM, allowing relevant differences between crops through crop-specific assignment of *C-*factors (see below). The UKCEH LCM 2015 at 25-meter raster [32; Table 1] classifies the land surface into 21 broad habitat types. However, in LCM 2015 all arable land is represented as a single class. To distinguish crop types, data were combined with UKCEH Land Cover *Plus* Crops 2016 –the earliest year available– which provides crop identities for vector polygons representing all agricultural fields in GB [33; Table 1] (“the crop polygon layer”), see, e.g., [31]. A list of crops present in Land Cover Plus Crops is found in Table 2. The crop polygon layer was converted to 25-meter cells and *snapped* to LCM 2015. Subsequently, in the combination map, the arable and improved grassland in the LCM were reclassified into the specific crops from the crop polygon layer (Table 2). This combined Land Cover Map (LCM+) was used as InVEST input.

**Table 2:** List of Land Cover classes included in combined Land Cover Map as combination of [32] and [33], with the 10 different agricultural crops and agricultural grassland distinguished in [33] plus woodlands from [32] for which periodic cover management (*C-*factor) and Erosivity (*R-*factor) were calculated. When pixels are labelled agricultural land by [33], the [33] label takes precedence over [32]. ‡To avoid NDVI interpretation error caused by non-green but viable perennial vegetation with potential full root systems capturing sediment, non-crop non-woodland vegetation is included as annual values (SI-1 Tables in S1 File). †Assumed perennial green.

LCM+ Category	Source	LCM+ Category	Source
**C -factors as periodic values (**equation 7)		**C-factors as annual values‡**	
Broadleaved woodland	[32]	Neutral grassland	All as [32]
Coniferous woodland	[32]	Calcareous grassland	
Winter Wheat	All as [33]	Acid grassland	
Summer Wheat		Fen, Marsh, and Swamp	
Winter Barley		Heather	
Spring Barley		Heather grassland	
Maize		Bog	
Oilseed rape		Inland rock	
Potatoes		Saltwater	
Field Beans		Freshwater	
Beet		Supra-littoral rock	
Other crops		Supra-littoral sediment	
Agricultural grassland^†^		Littoral rock	
		Littoral sediment	
		Saltmarsh	
		Urban	
		Sub-Urban	

*Cover management factors* (*C-factor*, Fig 1)*:* The cover management factor quantifies the effects of land cover, cropping practices, and other management techniques on soil erosion; a high *C-* or *P-*value implies high potential erosion. In most RUSLE calculations, e.g., [3,7,45], there is a single annual value for cover management and associated tillage per crop type (*C*_*i*_ and *P*_*i*_). Such parameter values are often simply copied between studies [3,49], with the danger that these values are inappropriate for other locations. In this analysis, we generated GB-specific values for the cover management factor based on observed soil cover fraction; similar to the method used to calculate nutrient retention capacity in [25]. As crops cover the soil variably through the year, we designed an observational approach that encompasses the *C-* and *P-*factors jointly as their individual effects cannot be readily distinguished. We call this combination the *C-*factor. We did this using the Normalized Difference Vegetation Index (NDVI) [3,50], derived from satellite imagery. Periods corresponded to the 16-day interval data frequency of the MODIS data of [44]. The *C-*factor values per gridcell *i* for interval *j* were generated following [50] and [25], who reasoned that the *C-*factor is exponentially and inversely related to NDVI:


$$\begin{array}{c} C_{ij}={exp}^{\left[-\alpha \left(\frac{{NDVI}_{ij}}{\beta -{NDVI}_{ij}}\right)\right]},\text{ where }\alpha \;=\;\text{2}\text{ and }\beta \;=\;\text{1}\text{ following }[\text{50}]. \end{array}$$ (7)

We used Copernicus Sentinel-2 NDVI data extracted via Google Earth Engine at 10-meter resolution, at 16-day intervals from 1 January 2016–31 December 2019. Multiple years were sampled to cover year-to-year variation. Available maps from the Sentinel-2 dataset were selected for the given 16-day interval, with an QA60 layer cloud mask set to no-data. The NDVI value taken was the maximum value found among gridcell NDVI scores within each 16-day interval. However, since these datasets did not provide full coverage, to cover missing areas we combined Sentinel-2 extractions with Terra satellite MODIS NDVI data for same 16-day intervals (MOD13Q1 v061) [44; Table 1], corrected for cloud cover (data were *resampled* to 25-meter resolution). The datasets were synchronised based on the ratio of overlapping gridcells: Sentinel-2 was recalculated to fit within the MODIS value distribution. Subsequently both datasets were averaged per gridcell.

**Fig 3:**
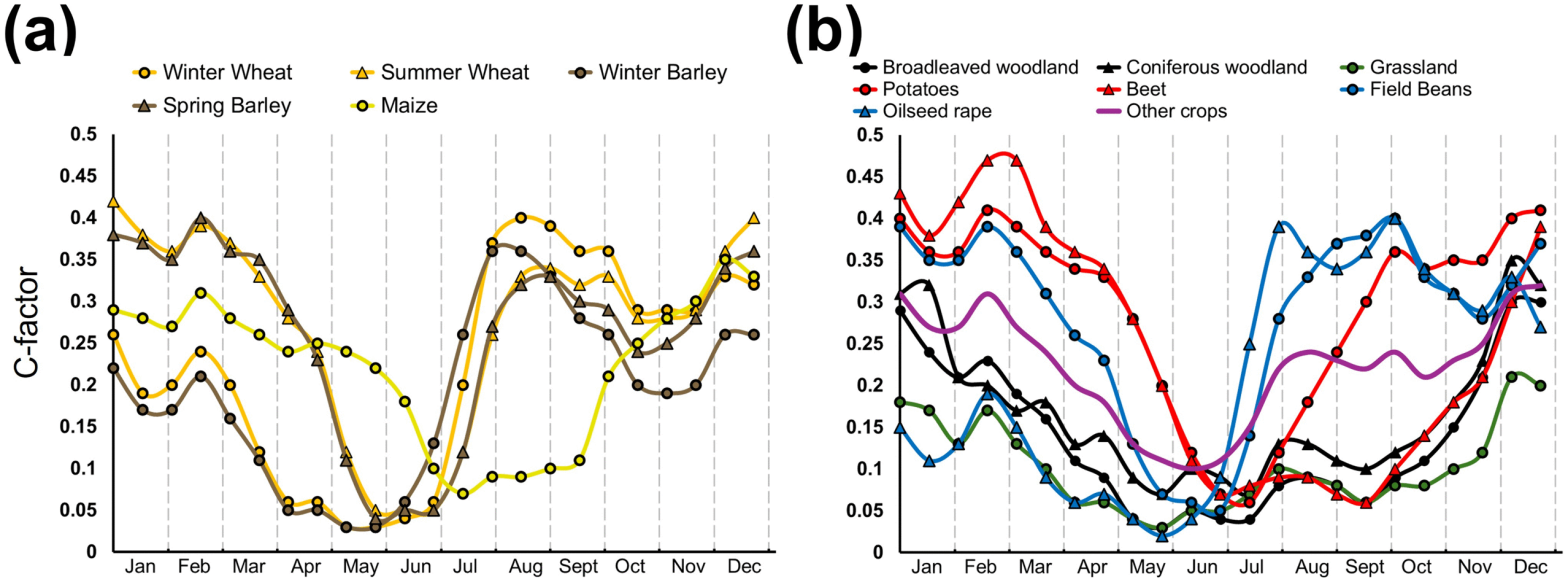
Crop specific cover management factors (*C-*factor) within GB over 16-days intervals calculated from observed NDVI for crops in [33]. (a) cereals; (b) non-cereal crops with woodland categories added from [32]. High *C-*factors indicate a high erosion potential, whereas low *C-*factors indicate a high capture potential of erosion by the vegetation.

The NDVI maps were converted to *C-*factor values for each gridcell. Subsequently, the mean *C-*factor value was extracted for each LCM+ class using the *Zonal* procedure, enforcing a 25-meter grid size. To cover year-to-year variation, values for each 16-day interval (e.g., 1^st^-16^th^ January; 17^th^ January to 1^st^ of February etc.) were averaged over the four years (2016, 2017, 2018 and 2019) per LCM+ class. The LCM+ classes cover ten crop types and (semi-) permanent agricultural grassland (Table 2). Fig 3 provides the estimates of crop specific cover management factors at 16-day intervals for the ten different crops and agricultural grassland, which is tabulated in SI-1 in S1 File. Fig 3 contains two deciduous woodland categories that fluctuate in cover through the year. To avoid NDVI interpretation error caused by misclassification as bare soil of periodically bare perennial vegetation with extant root systems that stabilise soils, non-crop non-woodland vegetation was included as single annual values (SI-1 Table in S1 File).

*Erosivity* (*R-*factor; SI-2 Fig in S1 File)*:* the erosivity index reflects the intensity and duration of rainfall affecting the amount of soil erosion. To allow for periodicity (*R*_*ij*_), we incorporated GB Gridded Estimates of Rainfall (CEH-GEAR) at a 1-km resolution [46; Table 1]. We used a period of 20-years (2000–2019) in combination with the regression approach of [51] (as reviewed in [3]). The longer period was to capture the substantial variation in annual rainfall, deriving to year-exceeding *R*-factor layers. Existing freely available erosivity data do not suffice for our periodic approach. For example, Panagos and co-workers [52] provide a complete rainfall erosivity map for the EU, UK (GB), and Switzerland based on the Rainfall Erosivity Database at European Scale (REDES), but only annually. Here, following [51], we converted CEH-GEAR with ≥10-mm rainfall per day to erosivity with their exact regression equation (adapted to 16-day intervals):


$$\begin{array}{c} R_{ij}=\text{6}.\text{5}\text{6}{Rain}_{\text{1}\text{0}(i,j)}-\text{7}\text{5}.\text{0}\text{9}{Days}_{\text{1}\text{0}(i,j)} \end{array}$$ (8)

where: *Rain*_*10(i,j)*_ is the summed 16-days rainfall for days with ≥ 10-mm rainfall for gridcell *i* and interval *j*. *Days*_*10(i,j)*_ is the number of days within an interval with ≥ 10-mm rainfall for gridcell *i* and interval *j* (the 10-mm threshold is as defined by [51].

These calculations were done individually for each 16-day interval over 20-years (2000−2019). Subsequently, to remove year-specific stochasticity values were averaged for each 16-day interval across all 20 years. We depict an annually summed *R-*factor map in SI-2 Fig in S1 File.

#### 2.3.2. Datasets and parameters from existing data sources.

*Soil erodibility* (K): the soil’s inherent susceptibility to erosion from rainfall and runoff per MJ mm^-1^ ha^-1^ erosivity. We used the soil erodibility map v.2014 at 500-meter resolution from the European Soil Data Centre [35; Table 1], *resampled* into 25-meter gridcells and subsequently *nibbled* to cover the full extent of GB, as set by the LCM + .

*Digital Elevation Map (DEM):* the UK Integrated Hydrological Digital Terrain Model at a 50-meter resolution [48; Table 1].

*Drainages (streams):* to let InVEST force streams calculated from the DEM match true observed patterns, the UK digital river centre-line network at a 1:50,000 scale was used [47; Table 1], *converted* to a 25-meter grid.

#### 2.3.3. Connectivity parameters.

*The threshold flow value* – the number of upslope pixels that must flow into a pixel before it is classified as a stream– was set at 100, after testing different values (10, 100, 250,1000) to visually match predicted streams to the drainage network of [47]. Examples are shown in SI-3 Figs and Text in S1 File.

*The Borselli IC*_*0*_ *parameter*, together with *k* below, represent calibration parameters that define the relationship between connectivity and the sediment delivery ratio (SDR, equation 3) [22]. *IC*_*0*_ was set at 0.47. This was estimated with a full year model run with default parameter 0.5 [22], following the resulting mean *IC* value was divided by the range and normalised.

*The Borselli k parameter* was set at 2, as recommended by [22]. Similarly, default NatCap recommendations were followed for the *maximum SDR value* (equation 3) that a pixel can have (set at 0.8), and the maximum allowed value of the *slope length parameter* (*L*) in the *LS* factor (set at 122).

### 2.4. Validation

#### 2.4.1. Observed sediment load.

The validation process is depicted in Fig 1. The validation data were location-specific concentrations of suspended solids (mg/l) reported in the water quality archive Water Information Management System (WIMS) [40; Table 1] for England; no similar Scottish or Welsh data were identified. Over the available 2013–2022 period the database category “Concentration of solids, suspended at 105 C” was selected for the monitoring type “River/Running surface water, sampled as periodic monitoring”. A total of 320 monitoring locations were selected based on the criterion of containing ≥ 5-years of data, and data from at least 10 samples.

As we needed a validation measure of total amount of sediment passing through the monitoring locations, we multiplied the WIMS-derived concentrations by multi-annual mean river discharge. For the latter, using the National River Flow Archive (NFRA) database of river flow discharges [41; Table 1], we selected the sediment monitoring locations that were within 1-km of a NFRA flow discharge gauging station (calculated using the *near* procedure in ArcGIS). The NFRA is the UK’s official record of river flow data, collating data from over 1,600 gauging stations all around the UK. We conducted manual checks to confirm closely matching WIMS and NFRA locations were on the same river course. The combined WIMS monitoring sites and NFRA gauging stations generated a validation dataset of 178 locations with both a multi-year mean of sediment concentrations and multi-year river discharge for the duration of measurements (Fig 2). A life-time mean concentration of suspended solids (‘sediment’) was calculated over all samples for each location. This as the within-year means would be based on a too highly variable number of samples, potentially skewing the distributions both within- and among-locations. Following [25], for every location the concentration of sediment (mg/l) was converted into metric tonnes of sediment flowing annually through the location, multiplying sediment concentrations with the annual discharge in litres: ‘the observed sediment load in streams’ (Fig 1).

#### 2.4.2. Modelled sediment load.

Using the delineation methods of [53], polygon catchments for the selected 178 locations were generated based on the same DEM (2.3.2). Subsequently, we summarised all realised erosion that reaches the streams (*E*) per polygon using the *Zonal* procedure following [53], iterating through the catchments to cover potential overlaps: ‘the modelled sediment load in streams’ (Fig 1). We enforced a 10-meter grid resolution in the *Zonal* procedure to minimise edge effects, while retaining consistency with the per hectare unit sum.

#### 2.4.3. Mathematical validation.

Both validation data per location (Observed sediment load year^-1^) and modelled data per catchment (modelled sediment load year^*-*1^) were divided by the area of the catchment. We normalised both datasets individually after logarithmic transformation (log_10_(*x*)+1). Following [24,53], to ameliorate the impacts of extreme values, we employed a double-sided Winsorising protocol for normalisation, using the 5% and 95% percentiles to define the 0 and 1 values (values below or above these percentiles became 0 or 1 respectively). See Matlab codes on GitHub. We employed two accuracy measures for model validation [24,53]: 1) Spearman’s *ρ*, comparing the rank order of predicted and validation data (Matlab *corr-*function); and 2) the inverse of the deviance (1-deviance) for ascertaining the absolute difference of each modelled value from its validation value. The latter accuracy measure ranges from 0 (no fit) to 1 (perfect fit = 100%). For interpretation of the inverse of the deviance, the criterion for AUC was employed, by which a result above 0.7 should be considered as significant [24].

## 3. Results

**Fig 4:**
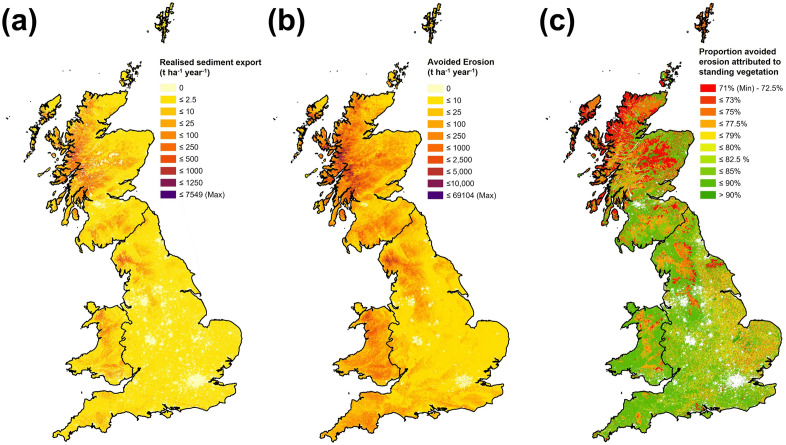
The GB wide estimate of realised erosion and erosion retention with a RUSLE mathematical approach using the InVEST modelling framework. Shown outputs from the developed model are as 50 x 50-meter with values in metric tonnes (t) ha^-1^ yr^*-*1^. (a) the estimated sediment exported per pixel that reaches the streams (*E*); (b) the avoided sediment erosion through retention by standing vegetation and in-field management (*V*); (c) the proportion avoided sediment erosion calculated per pixel ($\hat{V}$). UK shapes licensed under CC BY 4.0 [42].

We created a GB wide estimate of erosion and sediment retention for the year 2015, which we show in Fig 4. For more detail, in SI-4 Fig we depict the realised export proportionally per 16-day interval for England, Scotland and Wales, showing considerable within-year predicted periodicity with sediment export in winter much higher than in spring and summer.

*Realised sediment export that reaches the streams* (*E*; Fig 4a) was the highest when both erosivity was high and the relief (the variation in height and shape of the land surface) was substantial: such as in western Scotland, Wales, and Northwestern England. The total estimated annual export to streams is 24.5 million tonnes for England, 19.6 million tonnes for Wales, and 158 million tonnes for Scotland. Export to streams was strongly related to the relief slopes (Table 3). In which slopes above 10º (≈ 18 meters elevation difference per 100 meters slope length) account for only 15% of the research area but contain over 70% of all exported sediments. The 3.5% of area with the most slopes –above 20º– account for 50% of all erosion to the stream (Table 3). This makes model outcomes highly slope sensitive. Similarly, only 8% of the area has a rainfall erosivity (R-factor) over 500 [MJ mm^-1^] ha^-1^ yr^*-*1^, but that area accounts for 80% of the total export. The high rainfall erosive area coincides with the high relief areas in especially Western Scotland (SI-2 Fig in S1 File; correlation coefficient 0.55).

**Table 3:** Relationship between slope and exported sediments to the streams. Shown are slope classes generated from [48] and the mean exported sediments for these classes (total/area), in totals and proportions.

Slope Class	% of full area	Mean Exported t ha^-1^ y^-1^	Total Sediments to streams (10^6^ t year^-1^)	% of all export
No slope	5.1%	0.001	0.002	0.001%
≤1°	17.9%	0.015	0.062	0.03%
1° - ≤ 2°	16.7%	0.076	0.29	0.14%
2° - ≤ 3°	11.3%	0.30	0.76	0.38%
3° - ≤ 5°	15.6%	1.00	3.53	1.74%
5° - ≤ 10°	18.6%	4.96	20.9	10.3%
10° - ≤ 15°	7.6%	20.8	35.8	17.7%
15° - ≤ 20°	3.6%	49.4	40.3	19.9%
20° - ≤ 30°	2.7%	107	66.5	32.8%
30° - ≤ 75° (= max)	0.8%	185	34.5	17.0%

**Fig 5:**
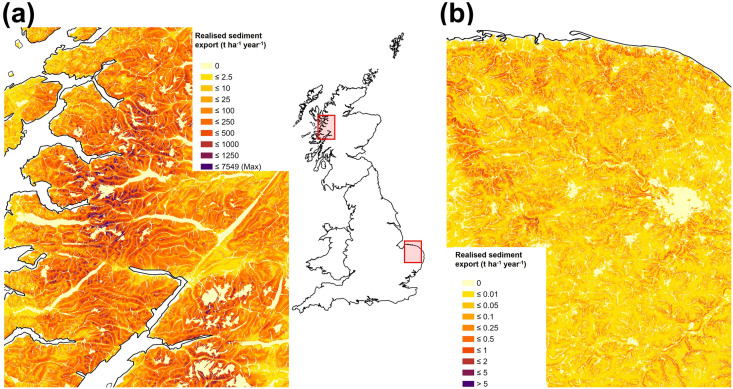
Details of the estimated sediment exported per pixel that reaches the stream (*E*) for (a) West-Scotland with high erosivity and high terrain and (b) East England, being flat and with substantially less erosivity. Note the change in scales between both figures. UK shapes licensed under CC BY 4.0 [42].

Differences in predicted erosion across GB are large, because of spatial variation in rainfall and terrain slopes. The median estimated export is (in t ha^-1^ year^-1^) is 0.03 for England, 0.88 for Wales and 0.98 for Scotland. However, export estimates are strongly skewed by small areas with high export. In high relief terrain in Scotland showed peaks in predicted erosion of >1000 t ha^-1^ yr^-1^ (Fig 5a; Table 3). Such peaks are likely anomalies found in very steep terrain, representing model-predicted rapidly growing gullies that can be detected in Fig 5a as purple stripes towards the streams. A more realistic 95% percentile value is a –still high– 321 t ha^-1^ yr^-1^ for this small Scottish area, indicating the steepness and complexity of the terrain (compare to Table 3 means). This equates to a soil layer of 3−4 cm thick being at risk in peak erosion areas annually (using 1 kg soil ≈ 1 litre). In contrast, in the low lying, flatter and generally drier landscapes of Eastern England – less rainfall erosivity and slopes–, sediment export was low. There, exported sediment did not exceed 5 t ha^-1^ yr^-1^, with a 95% percentile of 0.12 t ha^-1^ yr^-1^ for this area (Fig 5b, median 0.01 t ha^-1^ yr^-1^).

*Avoided erosion* (*V*; Fig 4b) was greater than the realised erosion by which sediment reaches streams: most potential erosion is captured by standing vegetation. Here, land cover classes with the lowest *C-*factors (Fig 3; SI-1 Tables in S1 File) such as woodlands, semi-natural vegetation, and crops with higher cover such as oilseed rape (which is green in winter) and Maize (in summer) had the highest avoided erosion. However, avoided erosion was highly related with the total amount of potential erosion and therefore followed the same general spatial patterns as realised erosion. To highlight the role of vegetation in capturing erosion, Fig 4c shows the proportion of erosion that was avoided ($\hat{V}$), which ranges from 70% to near 100% to avoided annually. Gully-prone heathlands and bog areas had low proportions of avoided erosion; *C-*factors of those land coverage categories are relatively high, indicating their high erosion potential (SI-1 Tables in S1 File). Grass-based agriculture showed high erosion avoidance compared to most crops, due to its year*-*round cover of vegetation. This difference between grassland and arable is the cause of the checkered patterns seen in east England and parts of east Scotland (Fig 4c), with areas that are predominantly crop-based exhibiting higher erosion (yellow and red colours) inter-mixed with lower-erosion grass patches (green colours). Nested within the crop*-*grass pattern, the ten crop categories themselves also led to variation, such as lower erosion avoidance with field beans and potatoes contrasting with winter wheat and winter barley (Fig 4c; the variation in non-green colouring). High resolution depictions are available at GitHub, with the GeoTiff layers at Zenodo.

**Fig 6:**
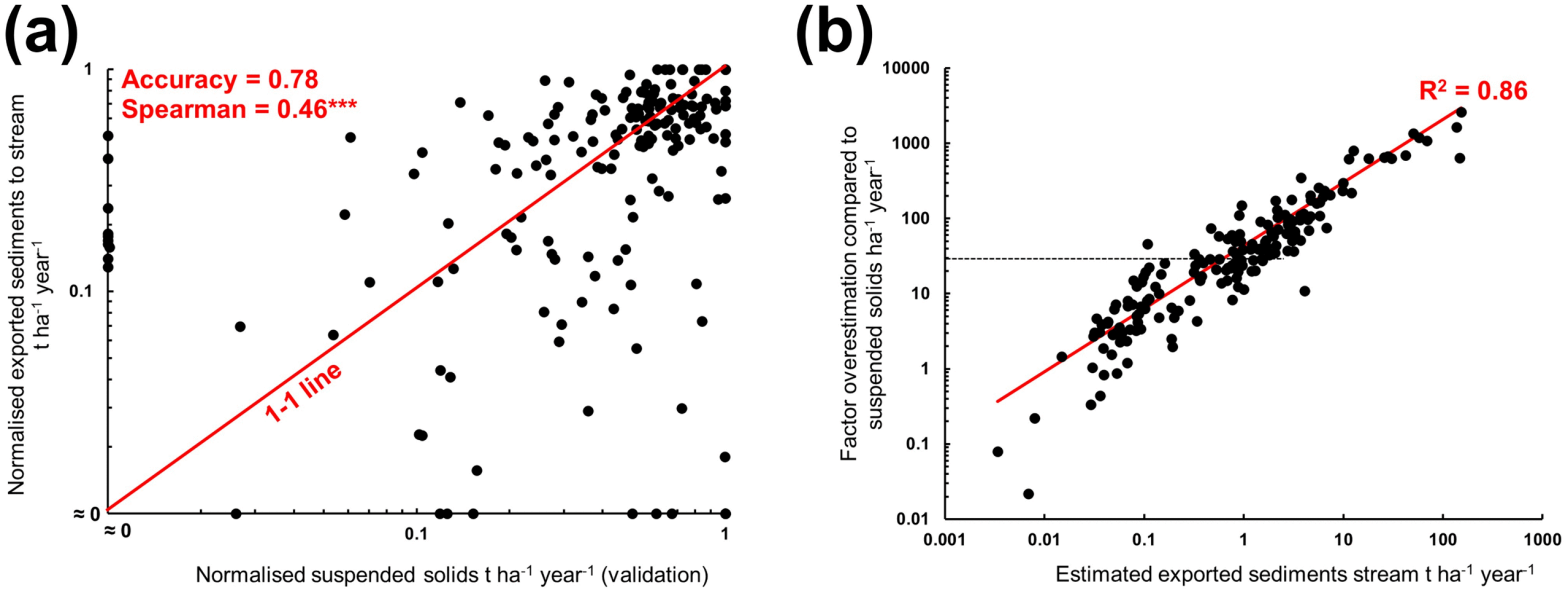
Correlations between observed sediment loads (validation) and predicted sediment export of the developed main model for 178 catchments, corrected for area. (a) plotted *relative* normalised (winsorisation protocol) validation *vs*. estimated amount of exported sediment. Note, logarithmic scales being used. (b) the relationship between the overestimation, in *absolute* numbers, of the developed main model compared to the validation set with a log-log regression (p < 0.001). With the magnitude of overestimation almost 1−1 correlated to the amount of estimated erosion [Log_10_(Overestimation) = 0.84xLog_10_(export) + 1.65]. The dotted line denotes the median factor 28 difference.

*Validation* (Fig 6) of the model resulted in a Spearman’s *ρ* of 0.46 and an accuracy of 78% (the inverse of deviance; Fig 6)*.* This indicates that the order of catchments was well-predicted and the model demonstrated good predictive power for *relative* values (NB, after normalisation; Fig 6a). For *absolute* values, we identified a median 28-times difference between observed sediment detected in rivers and modelled values for these 178 catchments (Fig 6b and compare scales of Figs 2 and 4). This model overestimation was logarithmically correlated to the amount of erosion per hectare, ranging from no overestimation at low predicted erosion to three orders of magnitude at high predicted erosion (Fig 6b).

*Parametrisation approaches*. We show a model improvement analyses for our new parameterisation approaches against generic model parametrisation runs (Table 4; methodological explanation in SI-5 Text in S1 File). Especially, the estimation of the required cover management factor based on satellite observations resulted in major improvements over generic value-transfer: an elevated Spearman *ρ* against validation data from 0.32 to 0.46 and deviance-based accuracy from 74% to 78% (Table 4). However, inclusion of these observed cover management factors also increased the prediction at least two-fold in tonnage (Table 4) as these C-factors are a factor two higher than that of generic value-transfer (SI-5 Table and Text in S1 File). Whereas the regression approach for the rainfall estimate (*R-*factor) did not improve or lower model accuracy compared to a more generic input, nor changes the estimated total export substantially (Table 4). Furthermore, adding periodicity had no effect over a full year average mode (Table 4)l: not only is total export highly similar, also the resulting values for the 178 validation catchments are near-perfectly correlated (R^2^ > 0.999; SI-5 Table in S1 File).

**Table 4:** Validation of the developed model in terms of accuracy (inverse of deviance) and Spearman ρ rank correlation against water quality monitoring data for sediments in rivers from [40] for model improvement runs. For comparison, a fully random model would result in an Inverse of Deviance of 0.5 and a Spearman *ρ* of 0. Provided are the summed exports the streams for the three countries in Great Britain (see Fig 2). ***p < 0.001.

	Inverse of deviance	Spearman ρ (ranking index)	Sediments to streams (10^6^ t year^-1^)		
			England	Wales	Scotland
**Developed main model**	**78%**	0.46***	**24.5**	**19.6**	**158**
**Model improvement tests (see also SI-5 Tabel and Text):**					
**1. No new approaches (generic baseline)**	74%	0.32***	12.4	10.8	124.4
**2. Not periodic with new C- and R-factors**	78%	0.46***	24.5	19.3	165
**3. New approach C-factor only (not periodic)**	77%	0.46***	29.2	27.6	222
**4. New approach R-factor only (not periodic)**	75%	0.35***	11.9	8.2	90.7

## 4. Discussion

We have presented a Great Britain-wide estimate of erosion and sediment retention with a Revised Universal Soil Loss Equation (RUSLE) model, using the well-established InVEST framework [Refs]. This work presents a parameterisation strategy for a data-rich area, which could be transferred to data-poor regions where validation is less possible. In normalised form the model output validated well against in-river suspended sediment, with a 78% accuracy on *relative* normalised data, which is better than expected following the review of model efforts in [5]: 63–69%, which included many RUSLE-type models. Our approach aimed to increase realism by adding a periodic cover agricultural management factor based on satellite observations resulting in substantial improvements in prediction accuracy.

### 4.1. Our approach to parameterisation

Our approach presents a distinct methodological advance in successfully applying the InVEST framework over a national extent. In itself, another RUSLE model of realised erosion is not a major novelty, as many such approaches exist, see [5]. However, our work serves as an example of how to parameterise the InVEST SDR model, enhancing its potential for observation-poor areas where formal validation is less possible, and with more seasonal rainfall. This supports the use of InVEST by decision makers with potentially limited modelling experience, for instance policymakers in the Global South [24]; as well as allowing for *in-silico* analyses of different management strategies and combinations of crops through comparing relative outcomes. Moreover, our approach is aimed at the GB as a whole, specifically at fine resolution, and thus can underpin analyses at a national level. Such approach could be combined with other more-generic modelled approaches, such as the EU-wide map of Panagos and co-workers [7]. Analyses on *relative* differences among locations could be used for prioritisation, targeting, or scenario development of erosion mitigation measures relevant to these predominantly agricultural landscapes in Great Britain.

We have demonstrated approaches to enhance the standard InVEST approach: a bespoke erosivity layer based on local rainfall data following [52], sub-annual periodicity, and cover management factors (*C-*factor) based on satellite observations (NDVI, following [25,50]. Evaluating these improvements separately indicated accuracy improvements are in majority achieved to the improved C-factors. Sub-annual periodicity did not yield different results than per year averaged input values, results are near-identical and fully correlated. As GB has year-round rainfall, periodic C-factor differences might even out. Though, when using this approach in seasonal rainfall climates, we foresee more merit of the periodic approach, which we suggest as strategy to incorporate in such areas.

The improved accuracy through more adequate C-factors is driven by the large differences among crop types, including that field beans and potatoes are generally less effective in reducing erosion, whereas winter*-*sown crops with standing vegetation early in the year (wheat, barley and oilseed rape) provide longer erosion protection compared to spring-sown crops. In our main model we use 4-year averages of C-values. In SI-5 Table and Text in S1 File, we show a substantial year-to-year variation as estimated cover differs. This year-to-year variation affects the *absolute* amount of sediments reaching the stream linearly –lowering of C-factors with 50% would reduce export estimates similarly (equation 1)–, but does not substantially alter the *relative* distribution of exported sediments as estimates are near-perfectly correlated (SI-5 Table).

The apparent good summer cover for maize requires a caveat, however. The NDVI is a canopy cover measure taken as a proxy for the amount of root; hence it does not consider that maize has an open sub-canopy and a shallow rooting system. Thus, surface erosion would be little hindered by lower layers, making the retention capacity of maize a likely overestimate. A next step could be to integrate below-canopy cover as a correction factor for the NDVI measure, for example through using multiple index regression techniques in combination with crop growth models [54].

The Land Cover Map Plus Crops for GB, available annually since 2015, allows one to distinguish among ten different crops plus agricultural grassland, and represent the different levels of retention they provide. Hence, the model benefits from not just land-use specific values but also from study-area specific values [28,29,49]. One of the problems normally faced when transferring parameters associated with land-use classes between studies is that numerous assumptions need to be made about the equivalence of different land cover schemes, such as calcareous and acid grasslands being equivalent to unmanaged grass, or heather and bogs being set as extensive grazing (SI-1 Tables; SI-5 Table in S1 File). An observational approach removes the need for such assumptions; at least for the arable categories where the vegetation is periodically removed, and in broadleaved forest where the foliage is shed. Therefore, we suggest incorporating and developing this satellite spectral observations in future RUSLE modelling. A caveat is that a periodic approach works less well in permanent cover vegetation, such as heathlands and semi-natural grasslands: here NDVI ‘confuses’ winter brown vegetation with bare ground: they reflect near-infrared and red light in similar proportions because of lack of active chlorophyll [55]. This elevates total estimated erosion by 10−15% depending on the area, especially outside of the growing season (SI-5 Table in S1 File). We suggest using annual averages for these vegetation types, as we have done here (SI-1 Table in S1 File).

In contrast, the new approach for the rainfall estimate (*R-*factor) did not improve or lower model accuracy compared to a more generic input (SI-5 Table in S1 File). Potentially, the regression equation used is a poor fit for GB, as the coefficients were trained on Iberian data [3,51]. It is beyond this study to train such a regression for the GB sites, for example with the REDES data-base, but we recommend this as a future avenue for exploration.

In general, we stress it is imperative for model accuracy to keep adapting methods [24,27], and generate inputs as close as possible to the equational assumptions. Therein, we emphasise the need for validation of models. Firstly, this provides an estimate of model ‘quality’ for the given region and time-period, similar to statistical testing for regular data [56]. Furthermore, validation metrics allow comparisons of uncertainty patterns among approaches, within and among studies [53], sensitivity analyses [29], and the attribution of uncertainty to environmental indicators. For example, Hooftman and co-workers [25] found that underperformance of flux-based algorithms correlated with the number of days that streams were frozen-over annually. Models such as InVEST are often applied in regions with limited observational or monitoring data, especially across much of the Global South [57]. In these contexts, models serve as substitutes for direct observation. Hence, validating models in data-rich regions like GB is a crucial to improve parameterisation, and build confidence in model performance when applied in data-poor regions where statistical validation is challenging. By contrast, attempting validation in regions with sparse or low-quality data may lead to misleading conclusions [24,58].

### 4.2. Validation of absolute amounts of erosion

On an *absolute* level, the match between modelled data and these observed validation data is poor, but explainable through differences in research objectives, discussed below. We found a median 28 times difference in absolute values between observed sediment (suspended solids) monitored in rivers and modelled accumulated realised erosion. This overestimation was correlated with the amount of modelled erosion per hectare, which makes it unlikely we interpreted an equation unit wrongly. A unit difference most likely would show as continuous error with variable noise, not as a strong exponential (log-log) directional correlation. There are no exponential calculations in RUSLE that could generate such patterns, as it uses a linear multiplication of factors (see equations 1 and 6). Moreover, our *relative* accuracy metrics compare normalised values. Hence, any unit misinterpretation is ruled out in our relative accuracy validation: which shows a good correlation (see above). In addition, individual deviances were not related to catchment size (linear regression: R^2^ < 0.01), i.e., the model does not consistently over*-* or underestimate in smaller catchments. Therefore, the normalised *relative* InVEST SDR model outputs are useful for policy and planning development such as for prioritisation of areas in terms of their comparative erosion levels [34,59] or *in-silico* scenario testing [60–62], whereas *absolute* numbers should be taken more care [3,20].

Absolute overestimations seem a general issue for such models as a similar overestimation was found for the EU-wide RUSLE output [7]. Against the same validation set, we calculate a median overestimation factor of 54 for [7], which is also logarithmically correlated to the amount of erosion per hectare (SI-6 Figs in S1 File), with a flatter slope because of less high peaks. This indicates that our parameterisation is not the primary source of absolute overestimation, but it points to systemic underlying issues. Though, there are substantial differences between both model outcomes: [7] tends to overestimate small amounts and large amounts of erosion with a same proportion, whereas our InVEST model skews especially higher erosion values upwards substantially (SI-6 Figs in S1 File), influenced by a high sensitivity to coinciding steep slopes and rainfall erosivity (SI-2 Fig in S1 File). Despite a lower median overestimation per validation catchment, more overestimation of high values leads a much greater summed total erosion of the InVEST model in comparison to [7] (SI-5 Table in S1 File), especially in rugged areas in Scotland and Wales.

First, with respect to the validation dataset, in-river measurements may not accurately capture the annual amount of erosion from the fields. The range of concentrations of suspended solids flowing through rivers per hectare catchment is much lower than the modelled range and lacks high values: the measured range is a factor 160 (maximum/minimum), compared to a factor 7,700 for our InVEST predictions (95% percentile/minimum). These in-river measurements are typically collected only few times per year. As erosion is a peak process [63], the likelihood that such peaks are captured in the validation data may be low. By contrast, in the model, realised erosion is computed using an erosivity factor, equated on the number of heavy rainfall events [3,7] and is therefore strongly peak driven. This contrast aligns well with the finding of better absolute validation on low predicted erosion levels (low peak flow) and the larger differences between measurements and predictions at high levels (high peak flow). This suggests a need for the development of monitoring schemes for in-river sediment that also sample or even focus on collection of sediment concentration data during peak flow periods.

Second, there is a difference in objective between RUSLE models and the data collection we used for validation. Both InVEST and [7] model erosion that reaches the streams and do not consider its fate further downstream, whereas the validation data is gathered in-stream. Therefore, in-stream sedimentation processes are not captured in these RUSLE models, which implicitly assume all sediment that reaches a stream is transported to the sea. In fact, most sediment will be deposited or captured by riverine vegetation [17]. We suggest that InVEST could incorporate an in-stream sedimentation function, echoing [25]. Since InVEST defines all watercourse pixels, a simple SDR*-*type approach – for instance, a reduction factor derived from stream slope and sinuosity– could be applied to approximate in-stream processes that accumulatively reduce the sediment load.

Third, RUSLE has been reported to overestimate, with the slope length-gradient factor (*LS*; equation 1) mentioned as a major problem [59,64,65]. The huge gully erosion peaks in Scotland of >1000 t ha^-1^ yr^-1^ are a likely product of this *LS*-factor issue and could be dismissed as anomalies. Although, these peaks are not dissimilar from similar InVEST estimated peaks reported in monsoonal Kashmir [66]], as annual rainfall peaks above 4000 mm in Scotland, with associated high erosivity (SI-2 Fig in S1 File). Because of the first mentioned issue, training the model on validation data is undesirable. Such will only accentuate the issue of insufficient range validation data, providing absolute predictions that are too low, as both we and [7] show. However, sensitivity testing this through the Borselli IC_0_ InVEST input parameters to which all calculated connectivity values and SDR-ratio are related [22], affects the total amount of estimated exported sediments to streams strongly (SI-5 Table in S1 File). Among others, we added a threshold value to indicate the effect direction (IC_0_ = 3), being potentially unrealistic [67] – a factor 6 above the indicated default value of 0.5 [22]–. Such high IC_0_ values lower the predicted values with a factor 3 (SI-5 Table in S1 File). However, on validation catchment level a median factor 9.5 overestimation remains. As well, adding a finer grained less rugged DEM lowers the total estimates with 20% –having reduced slopes (SI-5 Table and Text in S1 File). Altering the InVEST maximum length factor did not have a clear effect, suggesting short length slopes being responsible for high erosion estimates. These sensitivity analyses suggest the RUSLE *LS*-factor and associated connectivity only partly to be responsible for the overestimation. The issues mentioned above –lack of peak flow validation data, and no sedimentation function– likely have considerable impact on absolute validation.

Moreover, scale could play a substantial role in the absolute overestimation. Small-scale land uses such as field margins, shrubs and individual trees that are not included in the model’s land use type categories could be capturing substantial amounts of sediment [6]. Land cover maps including these features are being developed [68,69]. For now, in GB, our modelled country-wide patterns could be supplemented by investigating smaller, local areas using one of the many non-RUSLE models and associated local validation data [3,5,19]. This could also be combined with high precision Digital Terrain Model data, available at 1-meter scales in GB, and the newer version of the Land Cover Map, which is becoming available at 10-meters [69]. However, adding this further detail is difficult for data-poor regions.

### 4.3. Mitigation of potential erosion

We have shown through the development of NDVI-based *C-*factors that there are large and periodic among crop and vegetation differences in the proportion of avoided erosion. This information could be used to target erosion mitigation measures to crops and locations where they are most needed. The lowest proportion of avoided erosion was estimated for heathlands and bog areas, as those are mostly in more rugged areas with extensive livestock grazing. Here, engineering interventions that include dams and planting of buffer vegetation to avoid gullies could be beneficial [6,70]. By contrast, low-relief grass-based agriculture proved to be least susceptible to erosion, in agreement with previous research [71,72]. Although not-overgrazing and maintaining vegetation cover remain essential for stabilising the soil and securing water infiltration capacity [57].

Our findings suggest that both strategic crop*-* and cover crop selection may serve as an effective erosion mitigation approach. Fields vulnerable to substantial erosion from heavy rainfall events could benefit from increased planting of cover crops [6] and less disturbance from tillage [11]. Spatial diversification of crop types, combined with the adoption of appropriate tillage practices, may help prevent extensive surface sediment run-off [6,63]. Such practices are commonly featured within the frameworks of ‘conservation agriculture’ and ‘agri-environmental management’ [8]. *In-silico*, Schmaltz and co-workers [60] showed that erosion could be reduced with such measures when fields are exposed to environmental factors that elevate erosion (high erosivity and erodibility, high relief). In practise, positive effects are more difficult to assess but modelled results imply avoiding crops which leave bare soil in sloping terrain, such as potatoes and sugar beet, and reducing the exposure of soil with cover cropping. In addition, there are a range of novel technologies, including using geotextiles, plastics and rubber cover as mulches, chemical treatments as soil binders, soil netting, and laying of rock blankets [70].

### 4.4. Implications

The implications of this study are three-fold.

1) We present an example of improved parameterisation of modelling sediment erosion and erosion control of especially agricultural areas using the InVEST framework [22]. InVEST requires a low user’s knowledge – little mathematical or erosion-specific knowledge is needed–, making it less demanding to follow a parameterisation procedure.
2) We suggest improvements to national sediment load monitoring in rivers, by including measurements during peak flows, as currently sediment loads seem underestimated in the national Water Quality Archive data [40]. Not only would this provide better model validation data but also it would allow for better model parameter training through, e.g., AI techniques [73,74]. In combination, these first two suggestions should generate a greater confidence in modelling results and their usage in policy planning.
3) Gridcell-based maps with a complete national coverage, as for proportion avoided erosion in this work, are valuable for identifying and prioritising regions where more detailed assessment is needed to inform mitigation options [11,34,59]. Based on relative risk mapping policy-makers can identify which regions are more spatially homogenous in export patterns, where broad mitigation actions could be applied, and which areas are more heterogenous with more localised actions needed. Especially, this crop type-driven map would be great resource for such policy prioritisation and the recommendations of erosion countering Agri-Environment schemes [75]. Examples in GB include the English Environmental Land Management Schemes, Wales’s Sustainable Farming Scheme, or Scotland’s Agriculture Bill or in Europe under the EU’s Common Agricultural Policy (CAP).

## Supporting information

S1 File I1-6 File Supplementary Information 1–6 –containing SI’s 1–6. (DOCX)
