# Supplementary material for: Modification and validation of the InVEST erosion model for application at a national scale
Source: PLoS One. 2026 Jul 17;21(7):e0353736. doi: 10.1371/journal.pone.0353736 (PMC13379084; doi:10.1371/journal.pone.0353736)
Supplement: S1 File — (DOCX) [file pone.0353736.s001.docx]

**Supplementary Information 1-6**

**belonging to:**

**Modification and Validation of the InVEST Erosion Model for Application at a National Scale**

| **SI-1** | *C*-factor Tables | 1 |
| --- | --- | --- |
| **SI-2** | Utilised Erosivity factor and calculated Slopes | 3 |
| **SI-3** | Visual determination of most optimal Threshold Flow Accumulation input | 5 |
| **SI-4** | Periodicity by country in realised export | 8 |
| **SI-5** | New approaches and sensitivity analysis | 9 |
| **SI-6** | Validation of EU-wide RUSLE output of Panagos and co-workers for the same GB area against the same validation set | 16 |
| Supplementary Information References | | 19 |

**SI-1 *C*-factor Tables**

**Table SI-1-1.** **Utilised *C-*factors for 16-day periods** (combined *C* and *P* management factors, Eq. 1 & Eq. 6) calculated from observed NDVI (2016-2019) using [S1] for the crops in the Land Cover Plus Crops and woodland categories. These numbers are presented as graphical depiction in Figure 3 main text.

| **Calendar Day #** | **Broad’**  **wood** | **Conif’ wood** | **Winter Wheat** | **Summer Wheat** | **Winter Barley** | **Spring Barley** | **Maize** | **Oilseed rape** | **Potatoes** | **Field Beans** | **Beet** | **Other crops** | **Grass-land** |
| --- | --- | --- | --- | --- | --- | --- | --- | --- | --- | --- | --- | --- | --- |
| **1** | 0.29 | 0.31 | 0.26 | 0.42 | 0.22 | 0.38 | 0.29 | 0.15 | 0.40 | 0.39 | 0.43 | 0.31 | 0.18 |
| **17** | 0.24 | 0.32 | 0.19 | 0.38 | 0.17 | 0.37 | 0.28 | 0.11 | 0.36 | 0.35 | 0.38 | 0.27 | 0.17 |
| **33** | 0.21 | 0.21 | 0.20 | 0.36 | 0.17 | 0.35 | 0.27 | 0.13 | 0.36 | 0.35 | 0.42 | 0.27 | 0.13 |
| **49** | 0.23 | 0.20 | 0.24 | 0.39 | 0.21 | 0.40 | 0.31 | 0.19 | 0.41 | 0.39 | 0.47 | 0.31 | 0.17 |
| **65** | 0.19 | 0.17 | 0.20 | 0.37 | 0.16 | 0.36 | 0.28 | 0.15 | 0.39 | 0.36 | 0.47 | 0.27 | 0.13 |
| **81** | 0.16 | 0.18 | 0.12 | 0.33 | 0.11 | 0.35 | 0.26 | 0.09 | 0.36 | 0.31 | 0.39 | 0.24 | 0.10 |
| **97** | 0.11 | 0.13 | 0.06 | 0.28 | 0.05 | 0.29 | 0.24 | 0.06 | 0.34 | 0.26 | 0.36 | 0.20 | 0.06 |
| **113** | 0.09 | 0.14 | 0.06 | 0.24 | 0.05 | 0.23 | 0.25 | 0.07 | 0.33 | 0.23 | 0.34 | 0.18 | 0.06 |
| **129** | 0.04 | 0.09 | 0.03 | 0.12 | 0.03 | 0.11 | 0.24 | 0.04 | 0.28 | 0.13 | 0.28 | 0.13 | 0.04 |
| **145** | 0.03 | 0.07 | 0.03 | 0.05 | 0.03 | 0.04 | 0.22 | 0.02 | 0.20 | 0.07 | 0.20 | 0.11 | 0.03 |
| **161** | 0.05 | 0.10 | 0.04 | 0.05 | 0.06 | 0.05 | 0.18 | 0.04 | 0.12 | 0.06 | 0.11 | 0.10 | 0.05 |
| **177** | 0.04 | 0.09 | 0.06 | 0.05 | 0.13 | 0.05 | 0.10 | 0.09 | 0.07 | 0.05 | 0.07 | 0.11 | 0.05 |
| **193** | 0.04 | 0.07 | 0.20 | 0.12 | 0.26 | 0.12 | 0.07 | 0.25 | 0.06 | 0.14 | 0.08 | 0.15 | 0.07 |
| **209** | 0.08 | 0.13 | 0.37 | 0.26 | 0.36 | 0.27 | 0.09 | 0.39 | 0.12 | 0.28 | 0.09 | 0.22 | 0.10 |
| **225** | 0.09 | 0.13 | 0.40 | 0.33 | 0.36 | 0.32 | 0.09 | 0.36 | 0.18 | 0.33 | 0.09 | 0.24 | 0.09 |
| **241** | 0.08 | 0.11 | 0.39 | 0.34 | 0.33 | 0.33 | 0.10 | 0.34 | 0.24 | 0.37 | 0.07 | 0.23 | 0.08 |
| **257** | 0.06 | 0.10 | 0.36 | 0.32 | 0.28 | 0.30 | 0.11 | 0.36 | 0.30 | 0.38 | 0.06 | 0.22 | 0.06 |
| **273** | 0.09 | 0.12 | 0.36 | 0.33 | 0.26 | 0.29 | 0.21 | 0.40 | 0.36 | 0.40 | 0.10 | 0.24 | 0.08 |
| **289** | 0.11 | 0.14 | 0.29 | 0.28 | 0.20 | 0.24 | 0.25 | 0.34 | 0.34 | 0.33 | 0.14 | 0.21 | 0.08 |
| **305** | 0.15 | 0.18 | 0.29 | 0.28 | 0.19 | 0.25 | 0.28 | 0.31 | 0.35 | 0.31 | 0.18 | 0.23 | 0.10 |
| **321** | 0.21 | 0.23 | 0.29 | 0.29 | 0.20 | 0.28 | 0.30 | 0.29 | 0.35 | 0.28 | 0.21 | 0.25 | 0.12 |
| **337** | 0.30 | 0.35 | 0.33 | 0.36 | 0.26 | 0.34 | 0.35 | 0.33 | 0.40 | 0.32 | 0.30 | 0.31 | 0.21 |
| **353** | 0.30 | 0.32 | 0.32 | 0.40 | 0.26 | 0.36 | 0.33 | 0.27 | 0.41 | 0.37 | 0.39 | 0.32 | 0.20 |

**Table SI-1-2.** **Utilised non-crop *C-*factors (combined *C* and *P* management factors) calculated from observed NDVI** (2016-2019) using the equation from [S1] for the non-crop categories from [S2] as annual mean values – non-periodic to avoid noise caused by non-green but viable perennial vegetation with root systems capturing sediments. The parameter transfer values used in SI-5 are shown, used to detect the level of accuracy improvement of these observed *C-*factors. †Following [S3]; ‡we do not list the periodic values for these classes here, by they can be obtained from the author upon request.

| **LCM+ Category** | ***C-*Factor‡** | **Estimation details** | **For SI-5** | **Estimation details** |
| --- | --- | --- | --- | --- |
| **Neutral grassland** | 0.14 | Annual mean | 0.05 | Unmanaged grass† |
| **Calcareous grassland** | 0.13 | Annual mean | 0.05 | Unmanaged grass† |
| **Acid grassland** | 0.25 | Annual mean | 0.20 | Extensive grazing† |
| **Fen, Marsh, and Swamp** | 0.17 | Annual mean | 0.10 | Mean extensive grazing and water |
| **Heather** | 0.28 | Annual mean | 0.20 | Extensive grazing† |
| **Heather grassland** | 0.28 | Annual mean | 0.20 | Extensive grazing† |
| **Bog** | 0.27 | Annual mean | 0.20 | Extensive grazing† |
| **Inland rock** | 0 | Non erodible (*i.e.* no soil to erode)† | 0 | Non erodible† |
| **Saltwater** | 0 | Water is non erodible for sediments | | |
| **Freshwater** | 0 | Water is non erodible for sediments | | |
| **Supra-littoral rock** | 0 | Shoreline only, no sediments into streams | 0 | Non erodible† |
| **Supra-littoral sediment** | 0 | Shoreline only, no sediments into streams | 0 | Non erodible† |
| **Littoral rock** | 0 | Shoreline only, no sediments into streams | 0 | Non erodible† |
| **Littoral sediment** | 0 | Shoreline only, no sediments into streams | 0 | Non erodible† |
| **Saltmarsh** | 0.22 | Annual mean | 1 | Tidal flats [S4] |
| **Urban** | 0 | Non erodible† | 0 | Non erodible† |
| **Sub-Urban** | 0 | Non erodible† | 0 | Non erodible† |

**SI-2 Utilised Erosivity factor and calculated Slopes**

**
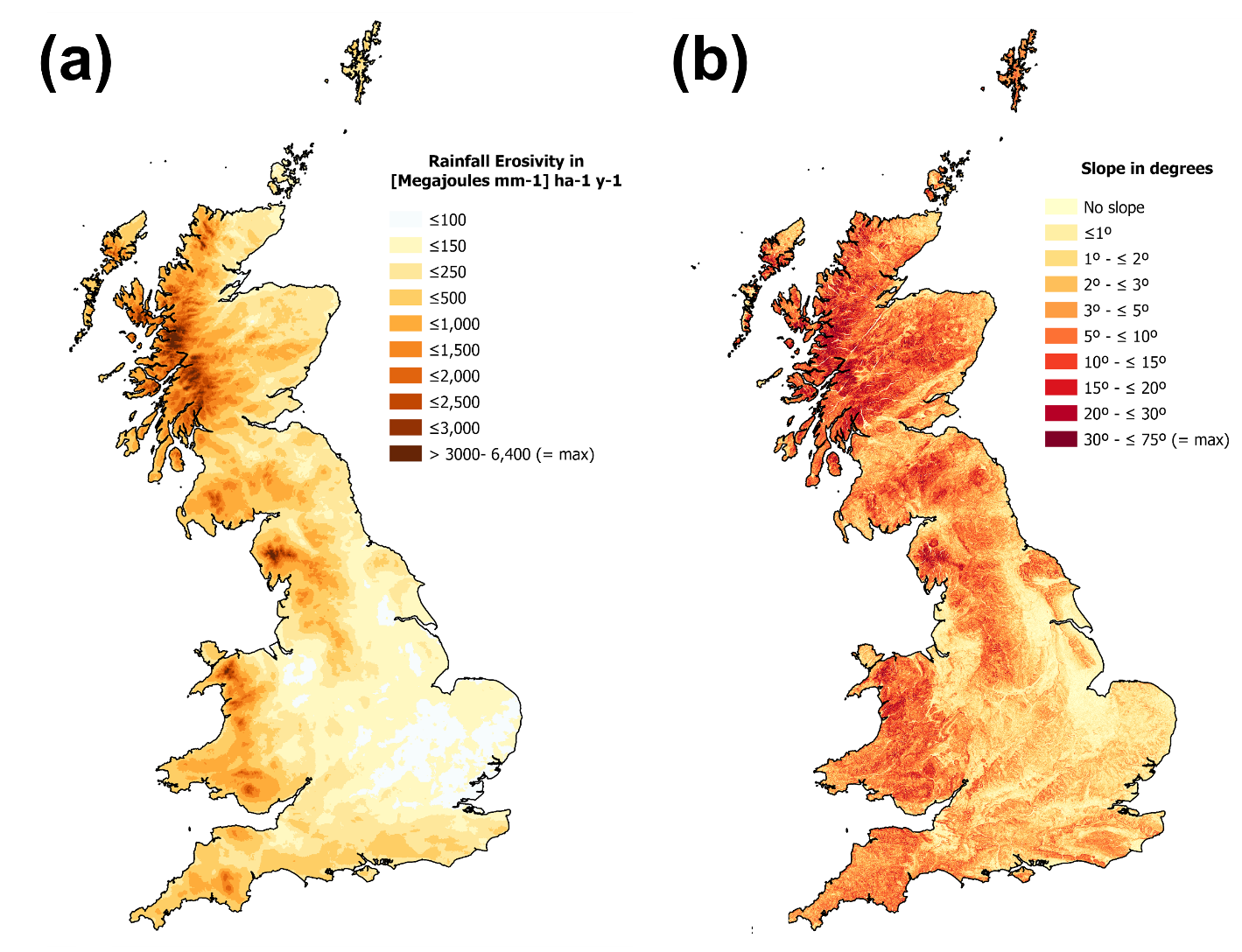
**

**Figure SI-2-1**. (a) Summed erosivity layer for GB, calculated annually from daily CEH-GEAR data [S5]; (b) Slopes as calculated from Morris & Flavin [S6,S7]. Areas of high values for both factors coincide: the Pearson correlation between both layers per pixel is 0.55. Note that calculation of both is independent, slope data are not part of the rainfall erosivity calculation.

**Table SI-2-1. Relationship between rainfall erosivity and exported sediments to the streams.** Shown are slope classes generated from [S5], see main text, and the mean exported sediments for these classes (total/area), in totals and proportions.

| **Rainfall erosivity in [MJ mm^-1^] ha^-1^ year^-1^** | **Area (% of full area)** | **Mean Exported t ha^-1^ y^-1^** | **Total Sediments to streams**  **(10^6^ t year^-1^)** | **% of all export** |
| --- | --- | --- | --- | --- |
| ≤100 | 7.7% | 0.03 | 0.06 | 0.03% |
| 100 - ≤ 150 | 23.8% | 0.10 | 0.54 | 0.27% |
| 150 - ≤ 250 | 25.0% | 0.73 | 4.17 | 2.06% |
| 250- ≤ 500 | 22.3% | 3.13 | 15.9 | 7.82% |
| 500- ≤ 1000 | 13.0% | 12.8 | 37.4 | 18.48% |
| 1000 - ≤ 1500 | 4.4% | 39.2 | 39.0 | 19.25% |
| 1500 - ≤ 2000 | 2.0% | 81.6 | 37.7 | 18.59% |
| 2000 - ≤ 2500 | 1.0% | 142 | 31.5 | 15.56% |
| 2500 - ≤ 3000 | 0.4% | 196 | 17.9 | 8.81% |
| 3000- ≤ 6400 (= max) | 0.3% | 291 | 18.5 | 9.14% |

**SI-3 Visual determination of most optimal Threshold Flow Accumulation input.**

The Threshold Flow Accumulation, a single value input in the model, is the number of upslope pixels that must flow into a pixel before it is classified as a stream based on the included DEM [S6, S7]. To determine the approximate best value, multiple thresholds were run for the model, with values 25, 100, 250 and 1000. As stream calculation is solely from the provided DEM [S8], all other inputs have no effect on the stream definition, neither has the presence or absence of periodicity. The outputs were visually compared to the in this paper used river definition network [S9]. Two depictions of separate areas are provided below (Figure SI-3-1).

We concluded that the InVEST calculated stream network was less complex than the actual network [S9], which is attributable to the used DEM [S8]. However, the extent of the river network, the location in space from were a gridcell was called a stream by InVEST, and so the stream starts, was best matched with [S9] by using the 100 Threshold value. The 25 threshold provides many superfluous and unrealistic parallel streams, whereas the 250 and 1000 even more provided a much more retracted steam network, i.e., streams would be estimated much later than suggested in [S9]. As the stream estimation is identical for every model run, using the [S6, S7] DEM, this value was kept constant throughout all calculation.


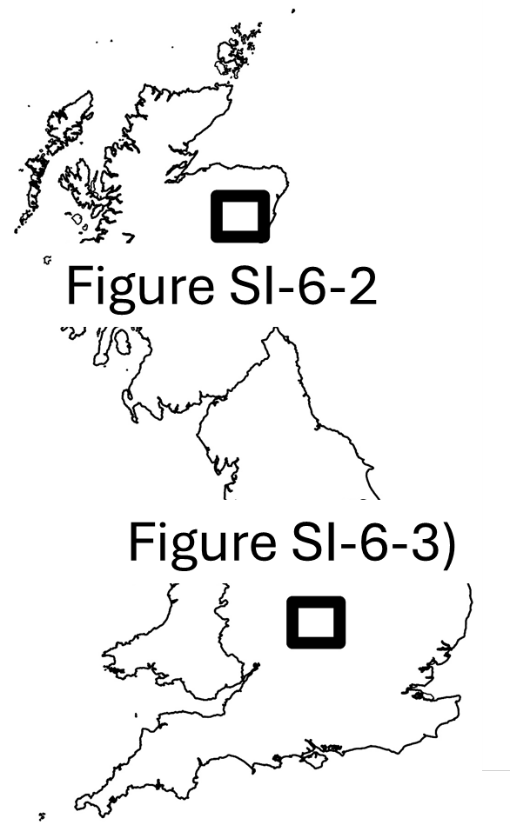


**Figure SI-3-1.** Location of the two areas for comparison of the Flow Threshold accumulation value.


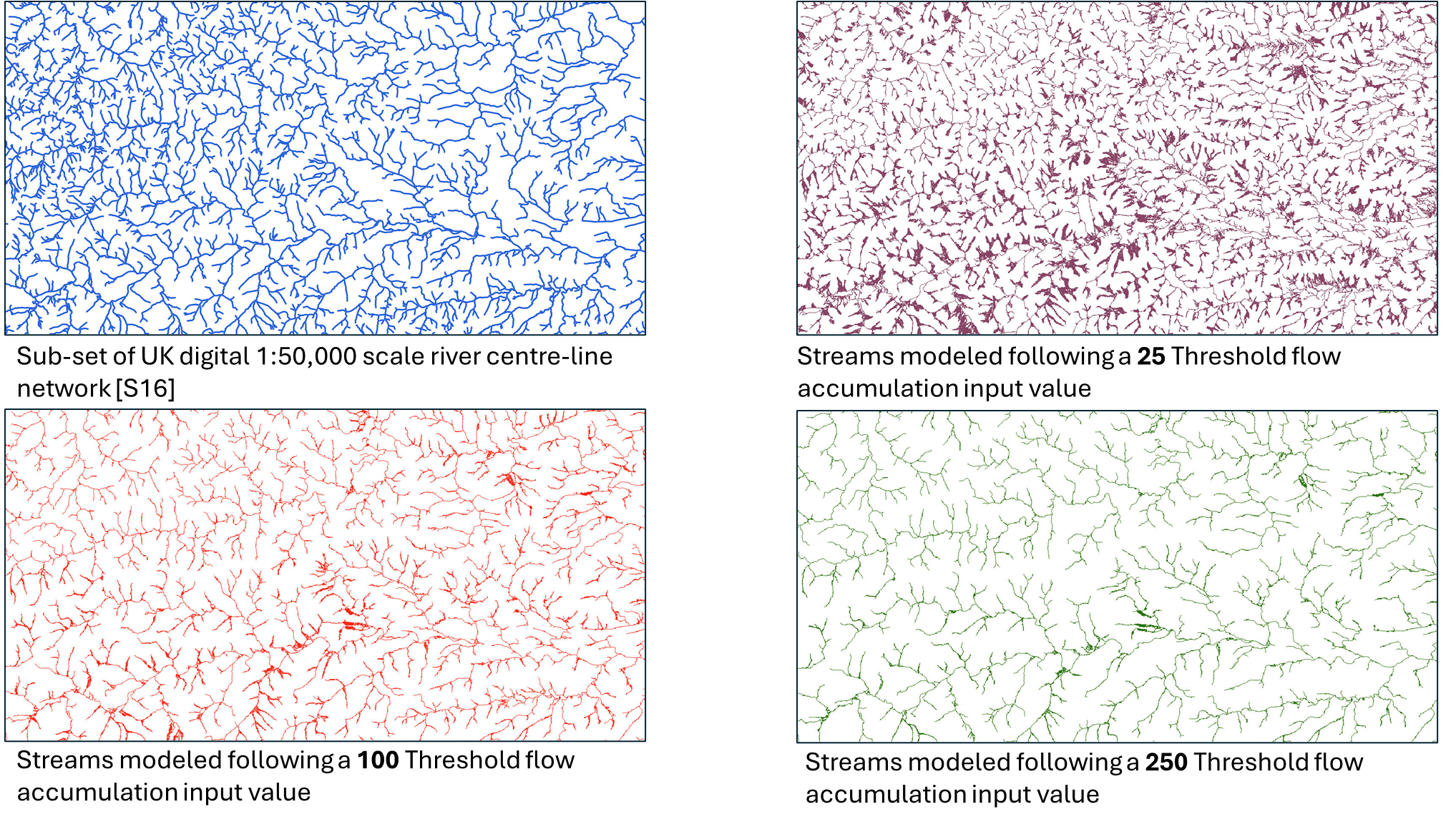


**Figure SI-3-2.** **Comparison of modelled stream rasters following 3 different Threshold flow accumulation factors for the first example area in Scotland**. Although less complex, the stream raster estimated with a 100 Threshold value matches visually best to the existing river centre lines [S9], whereas 25 brings much extra scatter and 250 is too restricted.

**
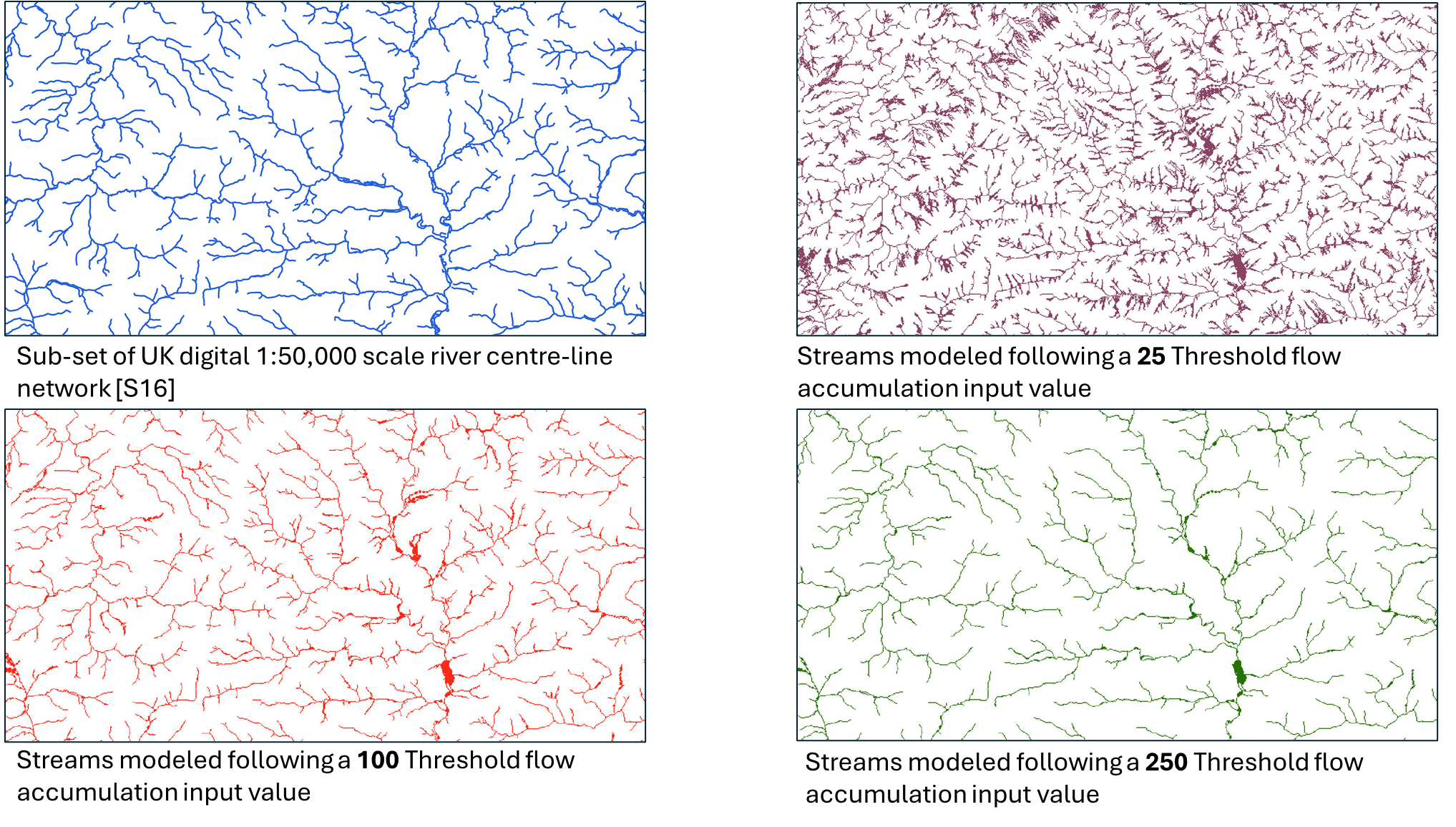
**

**Figure SI-3-3.** **Comparison of modelled stream rasters following 3 different Threshold flow accumulation factors for the second example area in middle England.** Although less complex, the stream raster estimated with a 100 Threshold value matches visually best to the existing river centre lines [S9], whereas 25 brings much extra scatter and 250 is too restricted.

**SI-4 Periodicity by country in realised export**

**Figure SI-4-1.16-day interval summed realised export to the streams per GB country**, as the proportion of annual export.

Total annual sediment export: Scotland: 158; Wales: 19.6; England: 24.5 (millions tonnes year^-1^).

**SI-5. New approaches and sensitivity analysis**

We evaluated whether the new approaches introduced in the paper provide an improvement over the standard methodology. The three new approaches are:

1. A periodic RUSLE model for InVEST. *Instead* of a single annual model (main text Eq.1 instead of Eq. 6).
2. Periodic GB-specific cover management *C*-factors for 11 crop types estimated using observed satellite Normalized Difference Vegetation Index (NDVI) values following [S1,S10]. *Instead* of a parameter transfer from earlier papers (Tables SI-1-2 and SI-5-2) [S3,S4,S11].
3. Periodic Erosivity maps (*R*-factor) following GB-specific Gridded Estimates of Areal Rainfall (CEH-GEAR) [S5] combined with the regression approach of [S12]. *Instead* of using the existing EU-wide map from Panagos and co-workers [S13].

To calculate any accuracy improvement for each of these three new approaches; four additional model runs were conducted with partially different data-sets, which were validated against the same 178 validation datapoints as in the main text [S14]. In Table SI-5-1, the accuracies of the four model improvement test are compared that for the main developed model as presented in the main text: the latter had a 78% accuracy (the inverse of deviance) and a Spearman *ρ of* 0.46 (Figure 6 Main text). Furthermore, the summed exported sediments are compared to investigate the effect of each approach on the total erosion estimate

Model improvement Test 1: a baseline set *without any new approaches*. This includes an annual model with a parameter transfer set –non NDVI based– for *C*-factors (Tables SI-1-2 and SI-5-2), no periodicity, and Erosivity from [S13]. I.e., including all ‘*insteads*’ above.

Model improvement Test 2: investigating the effect of *not-adding periodicity*. In an annual model, the new approaches for the *C*-factor and *R*-factor were included, but averaged into annual figures following $C_{i (annual)}=\frac{\sum_{j=1}^{23} C_{ij}}{23}$ and $R_{i \left( annual \right)}=\sum_{j=1}^{23} R_{ij}$.

Model improvement Test 3: investigating the effect of *NDVI based C-factors* *without adding both other new approaches*. In a non-periodic annual model, *C*-factors follow the NDVI-based annual mean values from Tables SI-1-2 and SI-5-2. Erosivity as from [S13].

Model improvement Test 4: investigating the effect of *GB-specific R-factors* *without adding both other new approaches*. In a non-periodic annual model, Gridded Estimates of Areal Rainfall (CEH-GEAR) [S5] combined with the regression approach of [S12]; the *C*-factors follow a parameter transfer set –non NDVI based– for *C*-factors (Tables SI-1-2 and SI-5-2).

Next to the above model improvement tests we conducted a series of sensitivity tests of differing InVEST inputs (except (2) being a different model). We validated each test against WIMS data [S14] and calculated the summed exported sediments for England, Scotland and Wales.

1. In the main model we set perennial vegetation classes as annual means also in the periodic calculations as NDVI calculations for non-green but viable perennial vegetation with root systems seem less not accurate, confusing brown vegetation to be exposed soil. As sensitivity test we have calculated those classes periodically and run the full main model.
2. The EU-wide map of assessment of soil loss by water erosion in Europe [S15; SI-6], which covers 81% of the research area but has limited coverage of high more upland erosion areas in bogs and marshes. Most likely since they would be labelled as non-erosive wetlands in the Corine land cover map used by [S15].
3. In the main text we used C-values based on a 4 year average 2016-2019, here we run them with C-values for the individual years (2016-2019), associated with the years specific CEH Land Cover® plus Crop Map [S16] and the respective NDVI values from [S17 & Google Earth engine]. These are in an annual model, the new approaches for the *C*-factor and *R*-factor were included, but averaged into annual figures and are therefore comparable to Model improvement test 2 – noticing that added periodicity has no added effect on accuracy or total summed sediment exported over the periodic model.
4. In the main text we state that the calculation of the LS-factor in RUSLE models is known for generating overestimates. Here we test the effect of several input parameters in an annual model comparable to Model improvement test 2 .

a./b. The IC-value which we set at 0 or at 1, instead of the 0.47 value as used in the main model. IC_0_ represents the transition point where the landscape's ability to trap sediment shifts to transport sediments into streams. In which a lower IC_0_ indicates more export. To indicate the direction of effect we also run the more unrealistic IC_0_ value of 3, which is the upper threshold border of acceptable values [S18]. This last run is seen as directional only but unrealistic in parameterisation.

1. The maximum length factor allowed by the model (see [S8]).
2. The DEM itself, by replacing the used 50m UK-DEM [S6, S7] by the 25m EU-DEM as used in [S15], running at 25m. Hence investigating the effect of a finer DEM resolution, potentially resulting in more realistic slope angles.

**Results**

*Model improvement tests:*

When comparing the developed model from the main text with the more basic ‘baseline’ model calculation without new approaches (Model improvement Test 1), the Spearman ρ rank correlation decreased from 0.46 to 0.32 (Table SI-5-1), *i.e.*, the catchment order match from low to high sediments per hectare catchments was substantially improved by our amendments. This Spearman rank improvement was caused by incorporating NDVI driven *C*-factors. However, there are a factor two difference in the total amount of expected exported sediments to streams between the developed main model and the baseline, the magnitude difference depending on the country. This is caused by adding calculated observed C-factors (Model improvement Test 3). The area-weighted mean of the C-factors across all land cover categories for the baseline is 0.084; whereas for the observed NDVI derived C-factors the area-weighted mean is 0.168. Higher C-factors linearly translated in higher erosion (see equation 1 main text). With the largest elevations in C-factor set in woodlands, agricultural grasslands, and the winter-open crops maize, summerwheat and spring Barley (See SI-1 and biophysical tables in SI-7).

Whether the model was periodic or non-periodic (Model improvement Test 2) led to neglectable differences, when still including new approaches for the *C*- and *R*-factors. The accuracy of the non-periodic and periodic runs were the same for both deviance based and ρ metrics (Table SI-5-1), with minor differences in total exported sediments for the different countries. When absolute per hectare values per validation catchment were correlated, there is a near-perfect correlation (R^2^ > 0.999; Table SI-5-1), indicating identical numbers in the same order.

When carried out as single new approach, adding observed *C*-factors resulted in a substantial better rank correlation compared to the baseline parameter-transfer model (Model improvement Test 3), and a slightly higher deviance-based accuracy (Table SI-5-1) – here the *R* layer was set as [S13] within an annual model. Furthermore, Model improvement Test 3 shows that an annual model with new approach *C*-factors and *R*-values from [S13] had approximately the same accuracy as the main developed model presented in the main text. However, as added above, the new C-factors resulted in higher erosion estimates because they are higher. Whether they are more realistic in their magnitude unknown since the validation values are 28 times lower within the validation catchments largely due to lack of peak-flow validation data.

Adding just GB-specific R-factors without adding the other new approaches led to no substantial improvement over the baseline model (Model improvement Test 4; Table SI-5-1). With the total amount of eroded sediments to the stream even tended to be lower. Hence this R-factor estimate might be less pessimistic, also compared to the EU-wide factors from [S15]. Potentially, the regression equation we used may be a poor fit for GB, as the coefficients were trained on Iberian data [S12,S19]. It is beyond this study to train such a regression for the GB sites, but this could be a future avenue of exploration.

**Conclusion of model improvement tests.**

**Adding observed NDVI-based *C*-factor values generated a substantial improvement over a standard model (baseline) in accuracy against this WIMS validation set [S14], while elevating estimated export.** Periodicity was not contributing to a better estimate, the accuracy was highly similar to the non-periodic model as were the total exported sediment totals with a near-identical correlation. As well, adding GB-specific *R*-factors did not result in an improvement in model performance.

*Further model sensitivity tests:*

Except for the EU-wide erosion estimate [S15] with a higher Spearman Rho (0.56), there are no differences in model accuracy among model sensitivity tests (test (2)). The difference between the developed model and the EU-wide model are shown in SI-6. Both models overestimate: the EU-wide model [S15] does overestimate compared to validation data with a more constant margin (Figure SI-6-2), leading the EU-wide model to overestimate more in low export conditions and our developed model to overestimate more in high export conditions (Figure SI-6-3). The outcomes of both models for the validation catchments are highly correlated (R^2^ = 0.71, with a 0.84 Person correlation)

All other sensitivity model runs are highly insensitive with respective to accuracy and ranking correlation, but not in absolute sediment export estimates. However, although the absolute numbers vary, all sensitivity runs are extremely highly correlated to the main developed model in the validation catchments calculated as tonnes export ha^-1^ y^-1^, indicating neither change in their order nor in relative difference in estimated exported erosion among catchments.

1. Including periodicity in permanent but temporally brown vegetation, potentially incorrectly indicated as open soil by NDVI, had an elevating effect on the total amount of exported sediments (Table SI-5-1). In the winter season, in which most erosion occurs (Figure 3 main text) , the estimated C values are higher because of the apparently lowered vegetation cover resulting in more erosion.
2. Using per year C-values instead of an average provided varying total amount of eroded sediments. The amount being correlated to the weighted mean of the per year C-values –i.e., lower C-values indicate a better retention capacity–. However, using per year values did not affect accuracy and years are near-identically correlated to the developed main model with 4-year averages. This indicates that using such an 4-year average might provide a good overall indication, being less subject to year-to-year variation
3. Changing the Borselli IC_0_ InVEST input parameter, to which all calculated connectivity values are related to [S8], affects the total amount of estimated exported sediments to streams. Lowering this value elevates the relative connectivity and so the total amount of sediments that can reach the stream, as shown by the estimates for IC_0_ = 0. (Table SI-5-1). Though without affecting the accuracy and with a near-identical correlation to the main developed model. In opposite elevating the IC_0_ value has the effect of lowering the relative connectivity and so decreasing the amount of sediments able to reaching the streams. This as shown by estimates for IC_0_=1, without affecting the accuracy and with a near-identical correlation to the main developed model. In addition we added a threshold value and so being potentially unrealistic [S18]– a factor 6 above the indicated default value of 0.5 [S8]–, to indicate the effect direction (IC_0_ = 3). Such unrealistically high IC_0_ values lowers the predicted values strongly. However, is still doesn’t come close to extrapolated validation values (on validation catchment level a factor 9.5 overestimation).

Altering the DEM to a more finer scaled one has a lowering effect on the total amount of exported sediments in all three countries. This is likely since the slopes become less sharp. The mean Terrain Ruggedness Index [S20] of the EU-DEM [S15,S21] is half of that of the UK-DEM ([S6, S7]; England 0.97 vs. 2.46; Scotland 2.24 vs. 4.80; Wales 2.07 vs. 4.81). This is calculated without resampling of the UK-DEM as that would alter ruggedness depending on the resampling technique – value transfer will elevate ruggedness as slopes will sharpen, whereas any recalculation resampling technique will smooth ruggedness by reducing slope angles through averaging.

**Table SI-5-1.** Validation of the developed model in terms of accuracy (inverse of deviance) and Spearman ρ rank correlation against water quality monitoring data for sediments in rivers from [S14] for model improvement and sensitivity runs. For comparison a fully random would result in an Inverse of Deviance of 0.5 and a Spearman *ρ* of 0. Provided are the summed exports the streams for the three countries in Great Britain (Figure 2 Main text). As well provided is the R^2^ of the linear regression [Y = βX] for validation catchments against the developed full model

|  | **Inverse of deviance** | **Spearman ρ (ranking)** | **Sediments to streams (10^6^ t year^-1^)** | | | **R^2^ to full model** |
| --- | --- | --- | --- | --- | --- | --- |
|  |  |  | **England** | **Wales** | **Scotland** |  |
| **Validation estimate, extrapolation based on area**‡ | | | **0.77** | ‡ | ‡ | SI-6 |
| **Developed main model (main text)** | **78%** | **0.46***** | **24.5** | **19.6** | **158** |  |
| **Model improvement tests:** | | | | | |  |
| 1. **No new approaches (baseline)** | 74% | 0.32*** | 12.4 | 10.8 | 124.4 | 0.87 |
| 1. **Not periodic with new C- and R-factors** | 78% | 0.46*** | 24.5 | 19.3 | 165 | > 0.999 |
| 1. **New approach C-factor only (not periodic)** | 77% | 0.46*** | 29.2 | 27.6 | 222 | 0.90 |
| 1. **New approach R-factor only (not periodic)** | 75% | 0.35*** | 11.9 | 8.2 | 90.7 | 0.96 |
| **Sensitivity runs:** | | | | | |  |
| 1. **All classes periodic (incl. brown vegetation)** | 78% | 0.47*** | 26.7 | 21.3 | 191 | 0.998 |
| 1. **EU-wide model [S15]†** | 78% | 0.56*** | 12.6 | 6.95 | 28.5 | SI-6 |
| 1. **Per year C-factors, not periodic with new C- and R-factors** (weighted mean 4-year average 0.17) | | | | | |  |
| 1. 2016 C-factors only (weighted mean 0.18) | 78% | 0.47*** | 27.0 | 21.2 | 169 | > 0.999 |
| 1. 2017 C-factors only (weighted mean 0.16) | 78% | 0.46*** | 22.6 | 18.2 | 146 | 0.998 |
| 1. 2018 C-factors only (weighted mean 0.16) | 78% | 0.46*** | 22.8 | 17.6 | 141 | 0.997 |
| 1. 2019 C-factors only (weighted mean 0.14) | 77% | 0.45*** | 19.5 | 15.5 | 125 | 0.998 |
| 1. **Inputs affecting the LS vector , not periodic with new C- and R-factors** | | | | | |  |
| 1. IC_0_ =0 | 78% | 0.47*** | 28.8 | 22.6 | 193 | > 0.999 |
| 1. IC_0_ = 1 | 78% | 0.47*** | 20.1 | 15.8 | 137 | > 0.999 |
| IC_0_ = 3 (for effect directionality only) | 77% | 0.48*** | 8.76 | 6.88 | 60.4 | 0.998 |
| 1. Maximum length factor (L) -25% & +25%^Ω^ | 78% | 0.46*** | 24.5 | 19.3 | 165 | > 0.999 |
| 1. EU-DEM 25meters as used by [S15,S21] | 78% | 0.46*** | 19.0 | 15.0 | 132 | 0.999 |

Table Notes: next page

Table Notes” *** P < 0.001; †Only 81% of the area coverage, through a Corine Land Cover based wider assessment of non-erosivity of surfaces, especially affecting rougher terrain in Scotland & Wales in which part bogs and heathlands are listed in Corine as non-erosive wetland. Linearly extrapolated to 100% coverage. ‡Only English validation data was used (see main text). Linearly extrapolated to full English area from 34% validation area coverage. Ω ≈ no difference with the full annual model (improvement test 2).

**Table SI-5-2.** Annual mean *C*-factor values, calculated from observed NDVI (2016-2019) and the parameter transfer *C*-factor values. Parameters following †[S3] ‡[S11]; ᵒ mean across all crops

| **LCM+ Category** | **Annual Mean (NDVI)** | **Parameter Transfer** |
| --- | --- | --- |
| **Broadleaved woodland** | 0.14 | 0.001 † |
| **Coniferous woodland** | 0.17 | 0.001 † |
| **Winter Wheat** | 0.22 | 0.10 ‡ |
| **Summer Wheat** | 0.28 | 0.10 ‡ |
| **Winter Barley** | 0.19 | 0.11 ‡ |
| **Spring Barley** | 0.27 | 0.11 ‡ |
| **Maize** | 0.22 | 0.20 ‡ |
| **Oilseed rape** | 0.21 | 0.08 ‡ |
| **Potatoes** | 0.29 | 0.34 ‡ |
| **Field Beans** | 0.28 | 0.32 † |
| **Beet** | 0.25 | 0.34 ‡ |
| **Other crops** | 0.22 | 0.26 ᵒ |
| **Grass-land** | 0.1 | 0.01 † |

**SI-6 Validation of EU-wide RUSLE output of Panagos and co-workers for the same GB area against the same validation set**


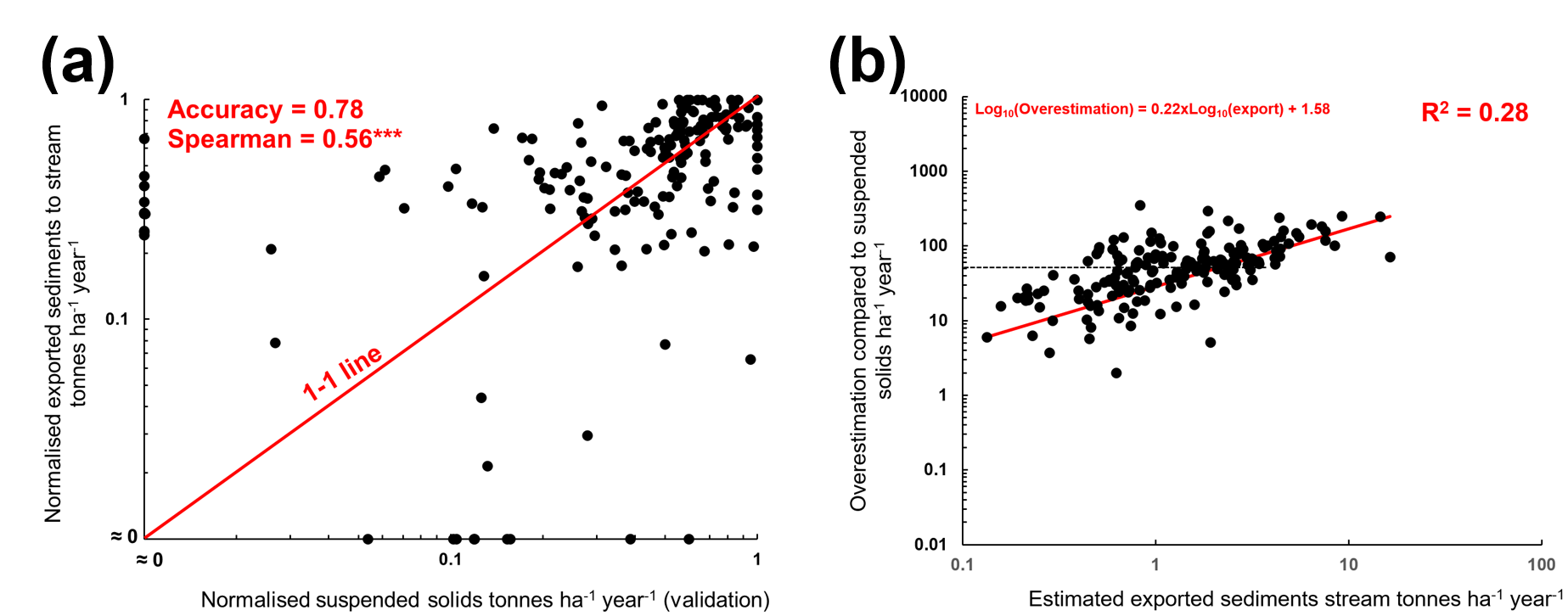


**Figure SI-6-1.** **Validation of the EU-wide model output of Panagos and co-workers [S15]**. Correlations of the sediment in rivers validation vs. the predicted amount of exported sediment from the developed main model for 178 catchments, corrected for area. (a) Plotted normalised (winsorisation protocol [S22]) validation vs. estimated amount of exported sediment. Logarithmic scales are used. (b) the relationship between the overestimation, in absolute numbers, of the developed main model compared to the validation set with a log-log regression (P < 0.001). Note the reduced slope compared to Figure 6 main text (0.22 vs 0.84), indicating [S15] has a lower value range compared to Figure 6, with consistently less peak predictions as well as less lower extremes. The dotted line denotes the median factor 54 difference.


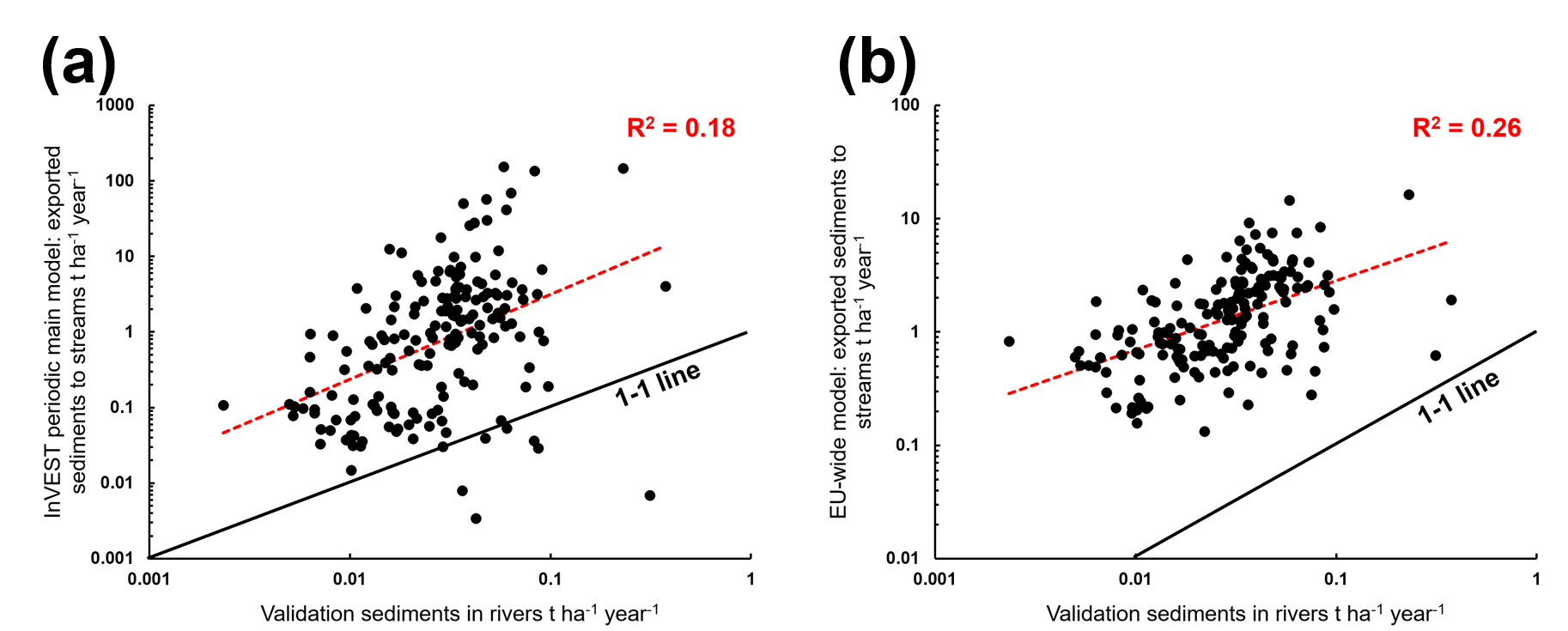


**Figure SI-6-2.** **Overestimations of the model output of (a) the main developed periodic model and (b) of the EU-wide model of Panagos and co-workers [S15]**. Correlations of the absolute amount of sediment in rivers validation vs. the predicted amount of exported for 178 catchments, corrected for area (total exported/hectares). Both models overestimate (above the 1-1 line). The EU-wide model (b; [S15]) does overestimate compared to validation data with a more constant but decreasing margin with increasing erosion (*β* = 0.61), whereas the InVEST model is more variable but has an increasing margin with increasing erosion (*β* = 1.12), i.e., the more erosion the higher the overestimation.


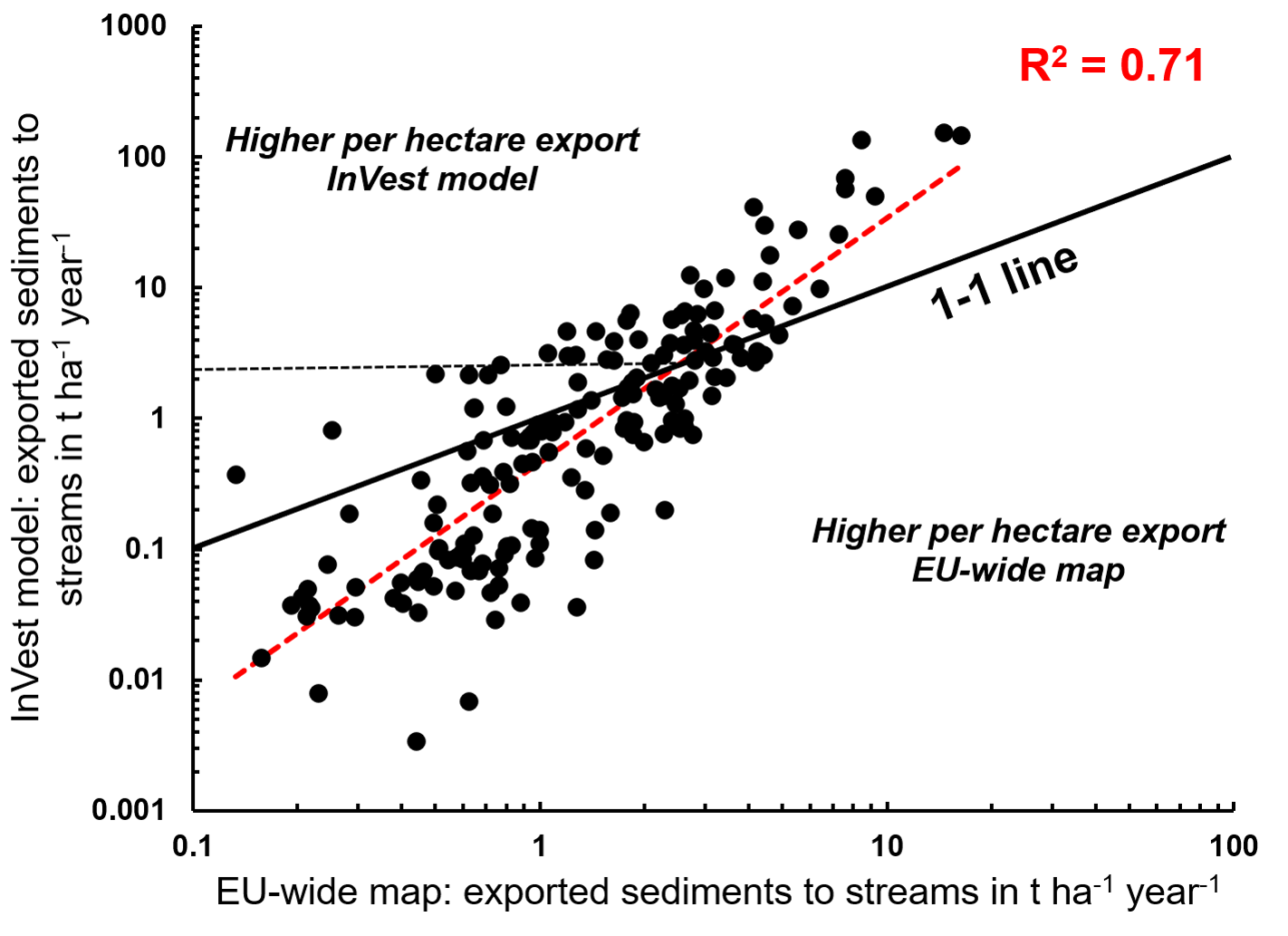


**Figure SI-6-3.** **Comparison between model output per validation catchment of the main developed periodic model vs. EU-wide model of Panagos and co-workers [S15]**. Both model outputs are highly but skewedly correlated ([Log_10_(InVEST) = 1.87xLog_10_(EU) – 0.33]. As a result, the EU-wide model of Panagos and co-workers [S15] has higher values below the cut-off point of 2.45 t ha^-1^ year^-1^, where the correlation (red line) crosses the 1-1 line (dotted line). Whereas above that cut-off point the InVEST model estimates higher values. This correlation results in a median overestimation that is higher of the EU-wide model (a factor 54) compared to the developed main model (factor 28). Note that such erosion estimates are not reached in the validation data set with a maximum value of 0.38 t ha^-1^ year^-1^. Because the overestimates at high values are more execurated by the main developed model (Figure SI-6-2) the total summed amounts across the full area is higher for the developed main model compared to the EU-wide model of [14], see Table SI-5-1, especially in more rugged areas such as Scotland.

**Supplementary Information References**

1. van der Knijff JM, Jones RJA, Montanarella L. Soil Erosion Risk Assessment in Europe. 2000. Available from: <https://www.unisdr.org/files/1581_ereurnew2.pdf>
2. Rowland CS, Morton RD, Carrasco L, McShane G, O'Neil AW, Wood CM. Land Cover Map 2015 (25m raster, GB). NERC Environmental Information Data Centre. 2017. Database. Available from: <https://doi.org/10.5285/bb15e200-9349-403c-bda9-b430093807c7>
3. Bakker MM, Govers G, van Doorn A, Quetier F, Chouvardas D, et al*.* The response of soil erosion and sediment export to land-use change in four areas of Europe: The importance of landscape pattern. Geomorphology 2008;98: 213–226.
4. Borrelli P, Robinson DA, Fleischer LR, Lugato E, Ballabio C, Alewell C, et al. An assessment of the global impact of 21st century land use change on soil erosion*.* Nat Commun 2017;8: 2013. Available from: <https://doi.org/10.1038/s41467-017-02142-7>
5. Tanguy M, Dixon H, Prosdocimi I, Morris DG, Keller VDJ*.* Gridded estimates of daily and monthly areal rainfall for the United Kingdom (1890-2019) [CEH-GEAR]. NERC Environmental Information Data Centre. 2021. Database. Available from: [doi.org/10.5285/dbf13dd5-90cd-457a-a986-f2f9dd97e93c](https://doi.org/10.5285/dbf13dd5-90cd-457a-a986-f2f9dd97e93c)
6. Morris DG, Flavin RW. *A digital terrain model for hydrology*. In: In: Brassel K, Kishimoto H, editors. Proc 4th International Symposium on Spatial Data Handling; Department of Geography, University of Zurich; 1990. pp. 250–262.
7. Morris DG, Flavin RW. Sub-set of UK 50 m by 50 m hydrological digital terrain model grids. NERC, Institute of Hydrology, Wallingford.1994, Database. Available from: [ceh.ac.uk/data/integrated-hydrological-digital-terrain-model](https://www.ceh.ac.uk/data/integrated-hydrological-digital-terrain-model)
8. Natural Capital Project (NatCap).  *InVEST 3.13.0. User Guide*. 2023. Available from: [storage.googleapis.com/invest-userguide/latest/en/](https://storage.googleapis.com/releases.naturalcapitalproject.org/invest-userguide/latest/en/sdr.html)
9. Moore RV, Morris DG, Flavin RW. Sub-set of UK digital 1:50,000 scale river centre-line network. NERC, Institute of Hydrology, Wallingford. 1994. Database. Available from: <https://www.ceh.ac.uk/data/15000-watercourse-network>
10. Hooftman DAP, Ziv G, Evans PM, Bullock JM. Validation of the InVEST nutrient retention model across Europe with attribution of model errors. Environ Model Softw 2025;106657. Available from: <https://doi.org/10.1016/j.envsoft.2025.106657>
11. Panagos P, Borrelli P, Meusburger K, Alewel, C, Lugato E, Montanarella L. Estimating the soil erosion cover*-*management factor at the European scale. Land Use Policy 2015;48: 38–50.
12. Ferreira V, Panagopoulos T. Seasonality of soil erosion under Mediterranean conditions at the Alqueva dam watershed*.* Environ Manag 2014;54: 67–83.
13. Panagos P, Ballabio C, Borrelli P, Meusburger K, Klik A, Rousseva S, et al. Rainfall erosivity in Europe. Sci Total Environ 2015;511: 801–814.
14. UK Data Service. *Water Quality Archive*. UK Department for Environment Food & Rural Affairs. 2023. Database. Available from:[environment.data.gov.uk/water*-*quality/view/download](https://environment.data.gov.uk/water-quality/view/download)
15. Panagos P, Borrelli P, Poesen J, Ballabio C, Lugato E, Meusburger, K, et al. The new assessment of soil loss by water erosion in Europe. Environ Sci Policy 2015;54: 438-447.
16. UKCEH. *CEH Land Cover® plus Crop Map*. UK Centre for Ecology & Hydrology. 2016. Database. Available from: <https://www.ceh.ac.uk/data/ceh-land-cover-plus-crops-2015>
17. Didan K. *MODIS/Terra Vegetation Indices 16-Day L3 Global 250m SIN Grid V061*. NASA EOSDIS Land Processes DAAC. Database. 2021. Available from: <https://doi.org/10.5067/MODIS/MOD13Q1.061>
18. Hamel P, Chaplin-Kramer R, Sim S, Mueller C, A new approach to modeling the sediment retention service (InVEST 3.0): Case study of the Cape Fear catchment, North Carolina, USA. Sci Total Environ 2015;524–525: 166–177.
19. Benavidez R, Jackson B, Maxwell D, Norton, K. A review of the (Revised) Universal Soil Loss Equation ((R)USLE): with a view to increasing its global applicability and improving soil loss estimates. Hydrol Earth Syst Sci 2018;22: 6059-6086.
20. Dilts TE, Blum ME, Shoemaker KT, Weisberg PJ,. Stewart KM. Improved topographic ruggedness indices more accurately model fine-scale ecological patterns. Landsc Ecol 2023;38 1395–1410.
21. European Environment Agency (2016). *EU-DEM (raster) - version 1.1.* [Data-Set] <https://sdi.eea.europa.eu/catalogue/srv/api/records/3473589f-0854-4601-919e-2e7dd172ff50>
22. Hooftman DAP, Bullock JM, Jones L, Eigenbrod F, Barredo JI, Forrest M, et al. Reducing uncertainty in ecosystem service modelling through weighted ensembles. Ecosyst Serv 2022;53: 101398. Available from: <https://doi.org/10.1016/j.ecoser.2021.101398>
